# *In-silico* prediction of RT-qPCR-high resolution melting for broad detection of emaraviruses

**DOI:** 10.1371/journal.pone.0272980

**Published:** 2023-05-08

**Authors:** Alejandro Olmedo-Velarde, Francisco M. Ochoa-Corona, Adriana E. Larrea-Sarmiento, Toufic Elbeaino, Francisco Flores

**Affiliations:** 1 Institute for Biosecurity and Microbial Forensics, Oklahoma State University, Stillwater, OK, United States of America; 2 Department of Entomology and Plant Pathology, Oklahoma State University, Stillwater, OK, United States of America; 3 Departamento de Ciencias de la Vida y de la Agricultura, Universidad de las Fuerzas Armadas ESPE, Sangolqui, Ecuador; 4 Istituto Agronomico Mediterraneo di Bari, Valenzano, BA, Italy; 5 Centro de Investigación de Alimentos, CIAL, Facultad de Ciencias de la Ingeniería e Industrias, Universidad UTE, Quito, Ecuador; Bangladesh Agricultural University, BANGLADESH

## Abstract

Twenty-four species of RNA viruses contain members infecting economically important crops that are classified within the genus *Emaravirus*, family *Fimoviridae*. There are at least two other non-classified species that may be added. Some of these viruses are spreading rapidly and cause economically important diseases on several crops, raising a need for a sensitive diagnostic technique for taxonomic and quarantine purposes. High-resolution melting (HRM) has shown to be reliable for the detection, discrimination, and diagnosis of several diseases of plants, animals, and humans. This research aimed to explore the ability to predict HRM outputs coupled to reverse transcription-quantitative polymerase chain reaction (RT-qPCR). To approach this goal a pair of degenerate genus-specific primers were designed for endpoint RT-PCR and RT-qPCR-HRM and the species in the genus *Emaravirus* were selected to framework the development of the assays. Both nucleic acid amplification methods were able to detect *in-vitro* several members of seven *Emaravirus* species with sensitivity up to one fg of cDNA. Specific parameters for *in-silico* prediction of the melting temperatures of each expected emaravirus amplicon are compared to the data obtained *in-vitro*. A very distinct isolate of the High Plains wheat mosaic virus was also detected. The high-resolution DNA melting curves of the RT-PCR products predicted *in-silico* using uMelt^SM^ allowed saving time while designing and developing the RT-qPCR-HRM assay since the approach avoided extensive searching for optimal HRM assay regions and rounds of HRM tests *in-vitro* for optimization. The resultant assay provides sensitive detection and reliable diagnosis for potentially any emaravirus, including new species or strains.

## Introduction

The genus *Emaravirus* (family *Fimoviridae*, order *Bunyavirales*) is a group of negative-sense ssRNA viruses created in 2011. *E*. *sorbi*, with European mountain ash ringspot-associated virus (EMARaV) as the member, was the first species characterized and classified in the genus [1, 2]. At present, twenty three other species are classified into this genus: *E*. *fici* (member: fig mosaic virus, FMV) [3], *E*. *rosae* (member: rose rosette virus, RRV) [4], *E*. *idaeobati* (member: raspberry leaf blotch virus, RLBV) [5], *E*. *cajani* (member: pigeonpea sterility mosaic virus 1, PPSMV1) [6], *E*. *tritici* (member: High Plains wheat mosaic virus, HPWMoV) [7, 8] which was formerly known as High Plains virus or wheat mosaic virus [9], *E*. *toordali* (pigeonpea sterility mosaic virus 2, PPSMV2) [10], *E*. *cercidis* (member: redbud yellow ringspot-associated virus, RYRSaV) [11], *E*. *actinidiae* (member: Actinidia chlorotic ringspot-associated emaravirus, AcCRSaV) [12], *E*. *rubi* (member: blackberry leaf mottle-associated virus, BLMaV) [13], *E*. *pistaciae* (member: Pistacia virus B, PiVB) [14], *E*. *parkinsoniae* (member: palo verde broom virus, PVBV) [15], *E*. *cordylinae* (member: ti ringspot-associated virus, TiRSaV) [16], *E*. *ziziphi* (member: jujube yellow mottle-associated virus, JYMaV) [17], *E*. *populi* (member: aspen mosaic-associated virus, AsMaV) [18], *E*. *perillae* (member: Perilla mosaic virus, PerMV) [19], *E*. *camelliae* (member: Camellia japonica–associated virus 1, CjEV1) and *E*. *verbanni* (member: Camellia japonica-associated virus 2, CjEV2) [20, 21], *E*. *syringae* (member: lilac chlorotic ringspot-associated virus, LiCRaV) [22], *E*. *kiwii* (member: Actinia virus 2, AcEV2) [23], *E*. *pyri* (member: pear chlorotic leaf spot-associated virus, PCLSaV) [24], *E*. *quercus* (member: common oak ringspot-associated virus, CORaV) [25], *E*. *aceris* (member: aspen mosaic-associated virus, AsMaV) [26] and *E*. *chrysanthemi* (member: Chrysanthemum mosaic-associated virus, ChMaV) [27]. Furthermore, two non-classified emaraviruses are alfalfa ringspot-associated virus (ARaV) [28] and pea-associated emaravirus (PaEV) [29]. Members of the genus *Emaravirus* infect important crops and can cause damage and economic losses in the countries where they are reported. The diseases, symptoms, economic impact, number of reported genomic segments, and the distribution of classified and non-classified emaraviruses are summarized in S1 Table.

Emaraviruses have a multipartite genome with four to ten genomic fragments [19, 30]. RNAs 1–4 are considered the core genomic RNA segments. RNA 1 encodes for the RNA-dependent RNA-polymerase (RdRp), RNA 2 for a glycoprotein precursor (GP), RNA 3 for a nucleocapsid protein (NC) and RNA 4 for a movement protein (MP) [2, 30, 31]. Whereas the RNAs 5 to 10, and in the specific case of PerMV named RNA 1–5, 6a-6c and 7, do not present clear homology between one another, and their protein-coded functions are yet to be determined [12, 19]. Furthermore, different emaraviruses are transmitted by different eriophyid mites (Subclass: Acari; Family: Eriophyidae) that can be transported long distances by strong wind [5, 8, 11, 32–36].

High resolution melting (HRM) analysis is a post-PCR technique that allows discrimination among species, isolates, and strains of viruses in a single reaction [37, 38], when coupled to dyes such as SYBR-Green or LC Green qPCR. It also has applications for single nucleotide polymorphism detection and genotyping studies [39, 40]. HRM exploits differences in the melting temperatures (°Tm) of PCR products.

After PCR amplification, the amplicons are melted, and the resulting changes in decreasing fluorescence are registered and plotted against the temperature increments. Once the melting temperature is reached, it results in unique melting curves obtained for each of the amplicons [37–39]. The method allows the detection and discrimination of individual amplicons among multiple targets, including primer-dimer formation and non-specific products. HRM coupled to qPCR has the potential to speed the diagnostic process since there is no need for electrophoresis [38, 40–42].

The RdRp viral genes are characterized by highly conserved regions. The highly conserved RdRp regions of emaraviruses are also present in the corresponding orthologs of members of the order *Bunyavirales* [30]. This prompted us to hypothesize the predicted melting temperatures *in-silico* of viral RdRp amplicons can be used to speed the design of oligonucleotide primer sequences for rapid development of HRM assays. Therefore, this project aimed to explore the application of the prediction *in-silico* of HRM outputs for speeding the development of RT-qPCR-HRM assays *in-vitro*, and second, to develop an RT-qPCR coupled to an HRM analysis system for emaraviruses for use in i) taxonomical studies; ii) detection and discovery of novel viruses, and iii) microbial forensics diagnostic tool to identify dissimilar and similar viral targets during biosecurity investigations.

## Materials and methods

### Viruses and infected plant materials

Symptomatic rose leaves infected with RRV, isolate OK-1, and buds of *Cercis* sp. infected with RYRSaV were collected in Stillwater, OK, USA. The tissue was transported refrigerated with ice. Plant samples were immediately used for nucleic acids extraction or stored at 4°C until used. Wheat leaves infected with HPWMoV, isolate 07–961, were kindly provided by Kansas State University, Hay Experiment Station (Dr. Dallas Seifers). Moreover, lyophilized infected plant tissues sourced from Agdia, Inc. (Elkhart, IN) were used as reference controls for HPWMoV, wheat streak mosaic virus (WSMV), Triticum mosaic virus (TriMV), Prunus necrotic ringspot virus (PNRSV), iris yellow spot virus (IYSV), groundnut ringspot virus (GRSV) plus tomato chlorotic spot virus (TCSV), tomato spotted wilt virus (TSWV), Impatiens necrotic spot virus (INSV) and maize stripe virus (MSpV).

### Primer design

RdRp sequences from AcCRaV, EMARaV, FMV, PPSMV1, PPSMV2, HPWMoV, RRV, RLBV, and RYRSaV were retrieved from the NCBI GenBank database. The last date of accession was January 17, 2016. A strand-sense normalization was performed due to inconsistencies in finding conserved regions if the sequences retrieved from GenBank were used directly in the sense they were deposited. The NCBI ORFfinder program (https://www.ncbi.nlm.nih.gov/orffinder/) was used to search for open reading frames (ORFs) coding for putative virus proteins and assessing whether the accessions were sense or anti-sense sequences. All negative sense sequences were aligned using MUSCLE [43] implemented in MEGA v.7.0.18. The selected gap open value was -450 [44]. Primaclade [45] was used for screening conserved domains and designing degenerate primers EMARA F7 and EMARA R8. The thermodynamic parameters of primers GC content, °Tm, and tendency to form secondary structures, were analyzed and studied *in-silico* with the assistance of Oligoanalyzer v3.1 [46]. The values of the thermodynamic parameters used for primer design were as described by [47]. The specificity of the primers was tested *in-silico* using Primer-BLAST [48]. The non-redundant (nr) database of GenBank was selected, last accessed on June 4, 2019.

### Viral RNA and total RNA extractions

Viral RNA was extracted from the RRV-infected rose leaves and RYRSaV-infected *Cercis* sp. buds following the protocol described by [49] and modified by [50] named direct antigen-capture method or direct trapping in plastic. The total RNA was extracted using the Rneasy Plant Mini kit (Qiagen, Valencia, CA) from lyophilized reference controls tissues carrying virus and wheat leaves infected with HPWMoV 07–961. The manufacturer’s instructions were followed with modifications described by [51].

### cDNA preparations

The synthesis of the first-strand cDNA was using random hexamer primers (12.5 ng/μL), 4 μL of RNA template (total RNA and plant viral RNA), and 200 U of Moloney murine leukemia virus reverse-transcriptase (M-MLV-RT) (Promega, Madison, WI) according to the manufacturer’s instructions for cDNA synthesis. The reaction was performed at 37°C for 90 min. Additionally, randomly-primed cDNA synthesized from plant tissues infected with EMARaV, PPSMV1, PPSMV2, and FMV isolates F3, dS, and 1B, were sourced from the Mediterranean Agronomic Institute of Bari (CIHEAM), Valenzano, Italy. The cDNA preparations were shipped at room temperature as isopropanol-precipitated nucleic acids. Resuspended cDNA was aliquoted and stored at -20°C until used.

### Optimization of the primers°Ta and Mg^2+^ concentration

All PCR assays were performed in reactions of 20 μL, and the cDNA of HPWMoV was synthesized from lyophilized tissue. Gradient PCR assays with annealing temperature (°Ta) ranging from 45.2°C to 65°C were performed to determine the optimum°Ta for the primer set EMARA F7/R8. Additional experiments were performed to determine the optimum magnesium (Mg^2+^) concentration from 1.5 mM to 5 mM. Serial dilutions of the template cDNA were also tested in combinations with the Mg^2+^ concentration to determine the best concentration and sensitivity.

### End-point RT-PCR

All the assays were performed using the optimized°Ta and Mg ^2+^ conditions in reactions of 20 μL, each consisting of 10 μL 2X GoTaq Green Master Mix (Promega), 1 μL of each 10 μM EMARA F7 and EMARA R8 primers, 2 μL of cDNA as template, 1 μL of 50 mM MgCl_2_ and 5 μL of DEPC-treated water. The cycling parameters consisted of an initial denaturation at 97°C for 2 min, followed by 40 cycles of denaturation at 95°C for 20 s, annealing at 45°C for 30 s and extension at 72°C for 30 s. A 3 min final extension was performed at 72°C. Either DEPC-treated water (non-template control, NTC) or cDNA synthesized from healthy plant tissue were included as negative controls in each PCR amplification experiment. All endpoint PCR amplifications were done in an Eppendorf 6321 thermal cycler (Eppendorf, Hauppauge, NY). The amplicons were visualized in a 2% agarose gel made in 1X TAE buffer and stained with SYBR Safe (Invitrogen, Carlsbad, CA). Product size migration was monitored using either 100bp or 1Kb plus ladders (Invitrogen, Carlsbad, CA).

### Two-step RT-qPCR-HRM with LC-Green

RT-qPCR-HRM reactions were in 20 μL volumes, each containing 10 μL of 2X Hot Start Master Mix (New England Biolabs, Ipswich, MA), 1 mL of each EMARA F7 and EMARA R8 primers (10 μM), 2 μL of cDNA as template, 0.9 μL of 50 mM MgCl_2_, 2 μL of 10X LC Green (Biofire Defense, Salt Lake City, UT) and 3.1 μL of DEPC-treated water. DEPC-treated water NTC was included in each experiment of RT-qPCR-HRM assay. Each reaction was repeated in triplicates. The qPCR cycling parameters consisted of an initial denaturation at 97°C for 2 min, followed by 35 cycles of denaturation at 95°C for 20 s, annealing 45°C for 30 s and extension 68°C for 30 s. A final 3 min extension was performed at 68°C. Post-qPCR HRM parameters used were pre-melting conditioning at 60°C for 1 min and a melting temperature cycle ranging from 60°C to 95°C, with increments of 0.5°C every 2 seconds. All RT-qPCR-HRM reactions were performed in a Rotor-gene 6000 thermocycler (Qiagen, Valencia, CA). Melting curve normalization was computer-assisted using Rotor-Gene Q-Series Pure Detection Software v. 2.3.1.

### Emaravirus inter-laboratory test

The inter-laboratory performance trial for primers EMARA F7/R8 was performed in two laboratories. The first trial was at Oklahoma State University, USA, and both endpoint RT-PCR and RT-qPCR-HRM were used. The cDNA preparations of HPWMoV, RRV, and RYRSaV were from the U.S., and were used in the first trial. EMARaV (isolate EM1), PPSMV1, PPSMV2, and FMV (isolates 1B, dS, and F3) were from Italy. DEPC-treated water was included as NTC.

The second trial was at CIHEAM, Italy, and only endpoint RT-PCR was tested. The two trials were spaced by three weeks. Replicate aliquots of the same cDNA samples were employed in the two trials. Plasmids containing the predicted diagnostic sequences of HPWMoV and RRV were used as positive controls. cDNA synthesized from RNA extracted from healthy fig leaves was included as a negative control. To confirm the broad detection of emaraviruses with the EMARA F7/R8 primers, RT-qPCR-HRM and endpoint RT-PCR assays were performed using cDNA of HPWMoV 07–961 and RYRSaV at different time frames at Oklahoma State University.

### Nucleotide sequence accessions and virus identity

The RT-PCR products amplified with primers EMARA F7/R8 were sequenced at the Oklahoma State University Core Facility. The BLASTn searches were performed against the viral database to validate the identity of the generated RT-PCR products. All the obtained diagnostic sequences were deposited in GenBank under accession numbers: KX397601–08.

### Positive controls and limit of detection

Positive controls were generated by cloning the partial RdRp gene sequences (306 – 309bp) of amplicons obtained from endpoint RT-PCR of HPWMoV, RRV, EMARaV isolate EM1 and FMV isolate 1B. The amplicons were gel excised, purified using the QIAquick Gel Extraction kit (Qiagen, Valencia, CA), cloned into a plasmid vector (pCR2.1-TOPO) using a TOPO-TA cloning kit (Invitrogen, Carlsbad, CA), and transformed into competent *Escherichia coli* cells using the One Shot Top10 kit (Invitrogen, Carlsbad, CA). The plasmids were purified from overnight grown bacterial cultures using a Plasmid Purification Mini kit (Qiagen, Valencia, CA), and bi-directionally sequenced at the Oklahoma State University Core Facility using the M13F and M13R primers.

The limit of detection (LoD) of the endpoint RT-PCR and RT-qPCR-HRM assays using primers EMARA F7/R8 were assessed using two types of templates, a positive control plasmid generated as described above and cDNA from HPWMoV 07–961. The plasmid concentration was determined by spectrophotometry using the NanoDrop v.2000 (Thermo Fisher Scientific, Inc., Worcester, MA). The plasmid concentrations ranged from 1 ng/μL to 1 fg/μL and were prepared by a ten-fold serial dilution. The unknown virus cDNA concentration of HPWMoV 07–961 was calculated from a plasmid standard curve for HPWMoV, following the method described by [51, 52]. DEPC-treated water was included as an NTC.

### Specificity assays

The specificity of the primers EMARA F7/R8 was assessed using endpoint RT-PCR. The reference positive controls tested were: the respective plasmids carrying the HPWMoV, RRV, and EMARaV amplicons, and cDNA synthesized from lyophilized reference controls for the rose-infecting ilarvirus PNRSV and the eriophyid mite-transmitted WSMV and TriMV. In addition, cDNA of phylogenetically related viruses with RNA genomes were tested; these were the orthotospoviruses IYSV, GRSV mixed with TCSV, TSWV, and INSV, and the tenuivirus MSpV. Healthy wheat leaves were used as healthy plant control and DEPC-treated water as NTC.

### Phylogenetic analysis

Due to low nucleotide sequence identity seen among emaraviruses, protein inferred sequences derived from nucleotide sequences of EMARaV isolate EM1, FMV isolates F3, 1B and dS, RRV, HPWMoV, HPWMoV 07–961, and RYRSaV were aligned using MUSCLE [43] with their respective homologs from GenBank, which was last accessed on September 01, 2019. The best model of protein evolution was used to generate a Maximum Likelihood (ML) tree with supporting bootstrap values following 1000 pseudo-replicates as implemented in MEGA v.7.0.18 [44].

### Prediction of melting temperature profiles

°Tm profiles were predicted for the RT-qPCR product sequences of HPWMoV 07–961, RRV, RYRSaV, EMARaV, and FMV (isolates 1B, dS, and F3) using uMelt^SM^, a web interface application developed for prediction of HRM and derivative profiles from nucleotide sequences [53]. The uMelt^SM^ parameters selected for *in-silico* prediction were 50 mM reaction molarity ([Mono+]), 3.2 mM free [Mg 2+], and 0% DMSO. The thermodynamic library was Blake & Delcourt 1998 [54]. The denaturing temperature range was from 65°C to 95°C with very high resolution (0.1°C). The value of the selected cooperativity factor (σ) was 0.184407. The σ value was determined by probing and comparing°Tm values *in-silico* and *in-vitro*. The σ factor defines the cooperativity of the system that enables the denaturation of DNA strands.

## Results

### Primer design and *in-silico* assessment of oligo sequences

A degenerate primer set, EMARA-F7/EMARA-R8, which targets partially conserved sites (S1 Fig), was designed to amplify a 306–309 bp product in the RdRp gene of emaraviruses. A 5’ sequence extension non-complementary to the targeted sequence was added to the primer EMARA R8. This six-nucleotide sequence extension is shown underlined and was added to equilibrate the°Tm and GC content of the two oligos as described by [47] (Table 1). Primers EMARA F7/R8 were found to be specific *in-silico* for 13 *Emaravirus* species after a Primer-BLAST search (S2 Table). This pairwise alignment included accessions of newly characterized emaraviruses. Primers EMARA F7/R8 amplify a product of 306 bp for EMARaV, FMV, PPSMV1, PPSMV2, RRV, PiVB, BLMaV, PVBV, RYRSaV, and AcCRaV, and 309 bp for HPWMoV, RLBV, and TiRSaV. In both cases, the non-complementary 5’ extension was part of the size of the amplicons. The thermodynamic features of primers EMARA F7/R8 are detailed in Table 1.

**Table 1:** Primer sequences of EMARA F7/R8 designed using Primaclade. Primer thermodynamic features were calculated using Oligoanalyzer v. 3.1.

Target virus	Primer name	Primer sequence 5’-3’	Length (bp)	Amplicon (bp)	°Tm (°C)	GC%	ΔG	ΔG self-dimer	ΔG hetero-dimer
*Emaravirus*	EMARA F7	TCTTGTGGTGATCCATGIARRCCYTTATTWCC	32	306 / 309	68.7–72.2	42	0.3	-14.19	-11.5
	EMARA R8	CCGCGCAGATAATCTTATARAIGAYAARYTRGAAT	35		66.4–71.5	36	1	-10.36	

ΔG values are expressed in kcal/mol and were calculated by Oligoanalizer V3.1. The underlined sequence of EMARA R8 is a 5´ sequence non-complementary to the targeted template (flap).

### Optimization of°Tm and Mg ^2+^ concentration

**Fig 1:**
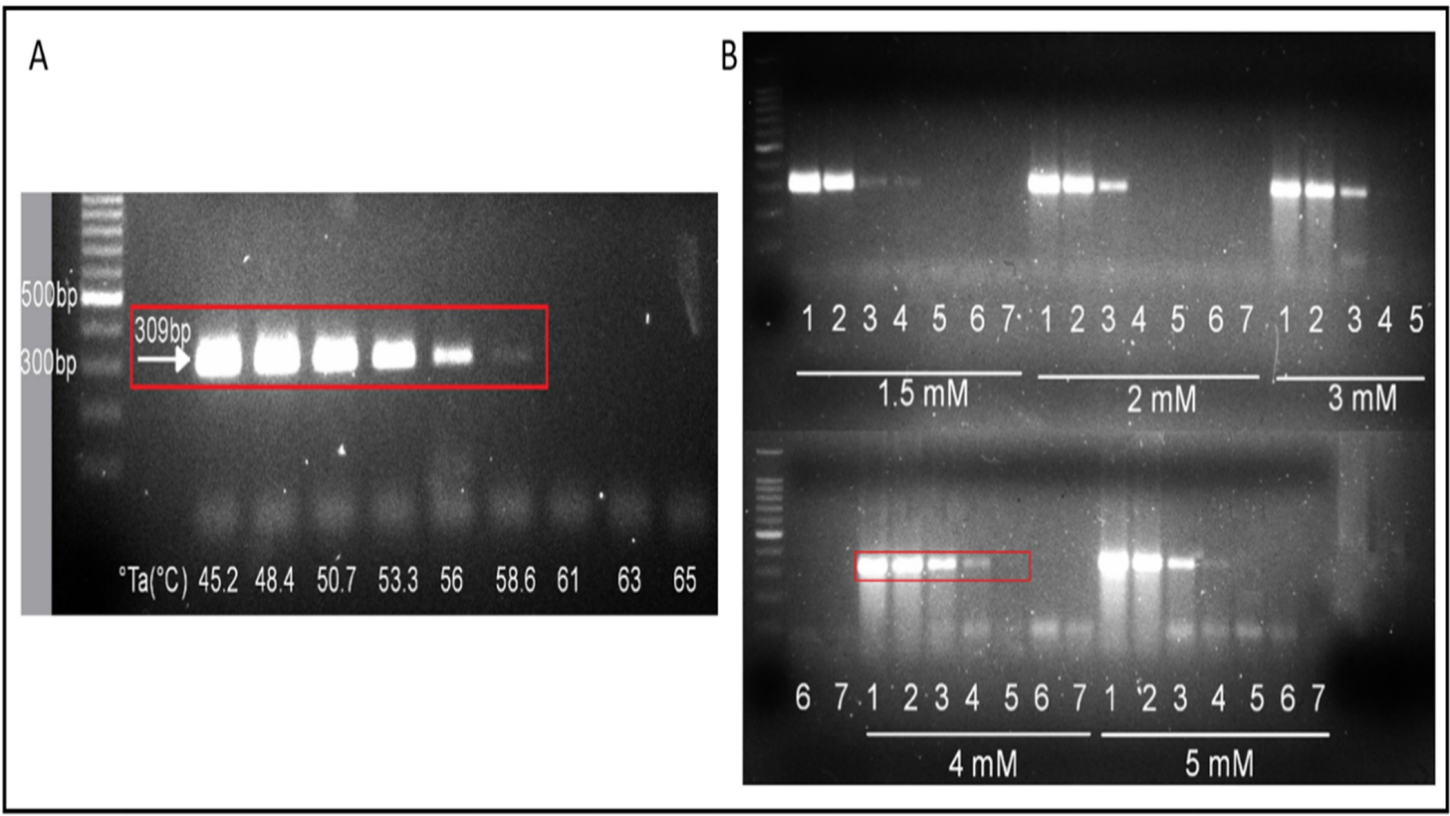
RT-PCR optimization using cDNA of High Plains wheat mosaic virus (HPWMoV). (A) Gradient PCR to determine optimum°Ta (45°C). (B) Assessment of Mg ^2+^ concentration and relative sensitivity to determine the optimal concentration (4 mM). Relative concentration of serially diluted cDNA: 1) 1 ng/μL. 2) 0.1 ng/μL. 3) 10 pg/μL. 4) 1 pg/μL. 5) 0.1 pg/μL. 6) 10 fg/μL. 7) 1 fg/μL.

The primer set EMARA F7/R8 amplified the expected product of the HPWMoV RdRp gene (306bp) within a range of six°Ta, from 45.2°C to 58.6°C (Fig 1A), with an optimum°Ta of 45°C. The best-obtained result of primers EMARA F7/R8 in a serial dilution showed the most brilliant product at the best relative sensitivity is shown in Fig 1B at 4 mM Mg ^2+^.

### Inter-laboratory detection of *Emaravirus* members

**Fig 2:**
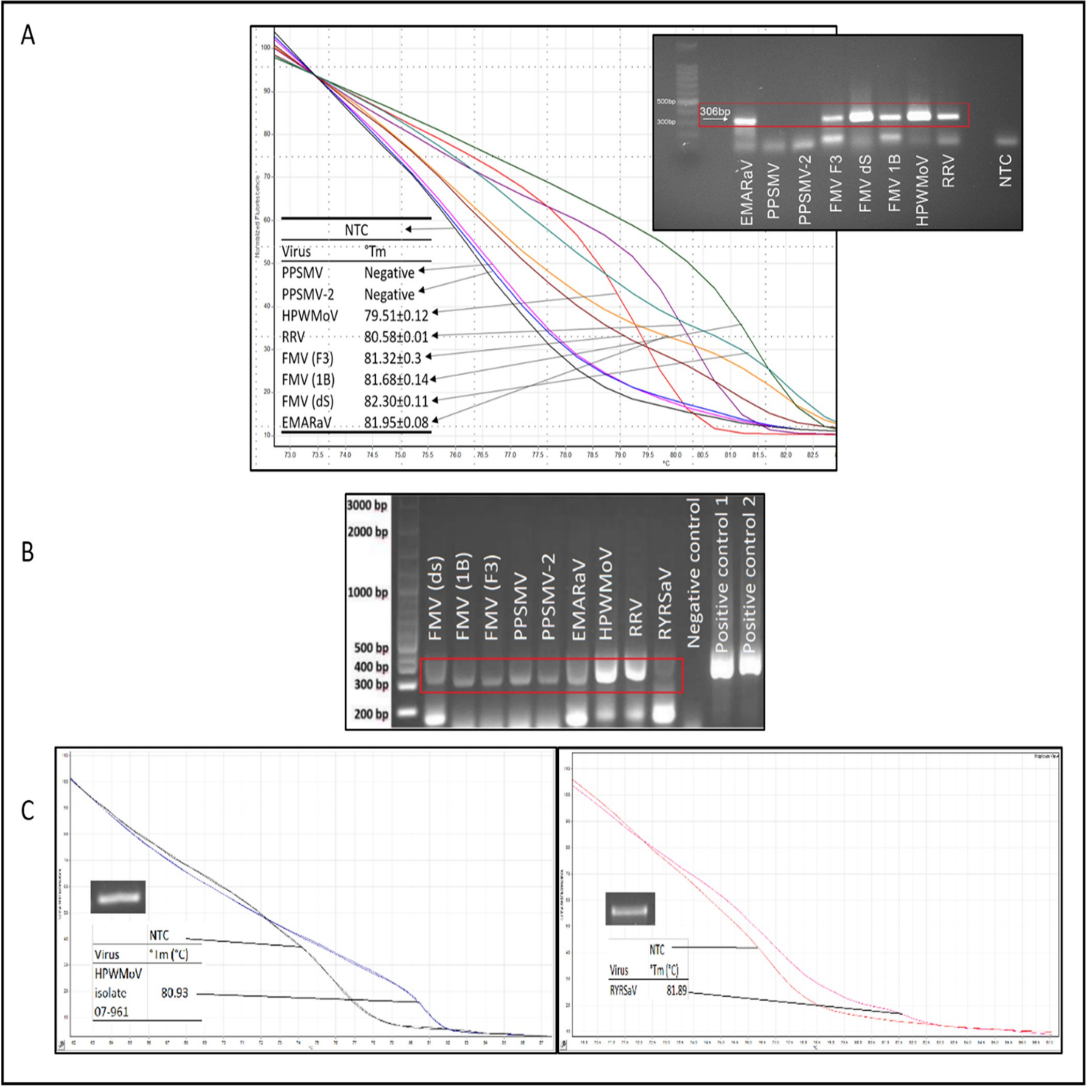
Multiple emaravirus detection assays. (A) First assay, RT-qPCR-HRM normalized melting curve showing species-specific patterns, and corresponding endpoint RT-PCR products in agarose gel electrophoresis for European mountain ash ringspot-associated virus (EMARaV), fig mosaic virus (FMV) isolates dS, 1B and F3, rose rosette virus (RRV), High Plains wheat mosaic virus (HPWMoV), pigeonpea sterility mosaic virus 1 (PPSMV1), PPSMV2 and non-template control (NTC). Both PPSMV1 and PPSMV2 were not detected in this assay. (B) Second assay, RT-PCR detection of FMV isolates dS, 1B and F3, PPSMV1, PPSMV2, EMARaV, HPWMoV, RRV, and redbud yellow ringspot-associated virus (RYRSaV). Positive controls 1 and 2 are plasmids harboring diagnostic sequences for RRV and HPWMoV, respectively. The negative control is cDNA of healthy fig tissue. (C) Third assay, RT-qPCR-HRM, and endpoint RT-PCR detection of HPWMoV 07–961 (left) and RYRSaV (right). RT-PCR detection products in an agarose gel are featured left-center of each plot. Detection assays were performed at different time frames and in different laboratories.

**Fig 3:**
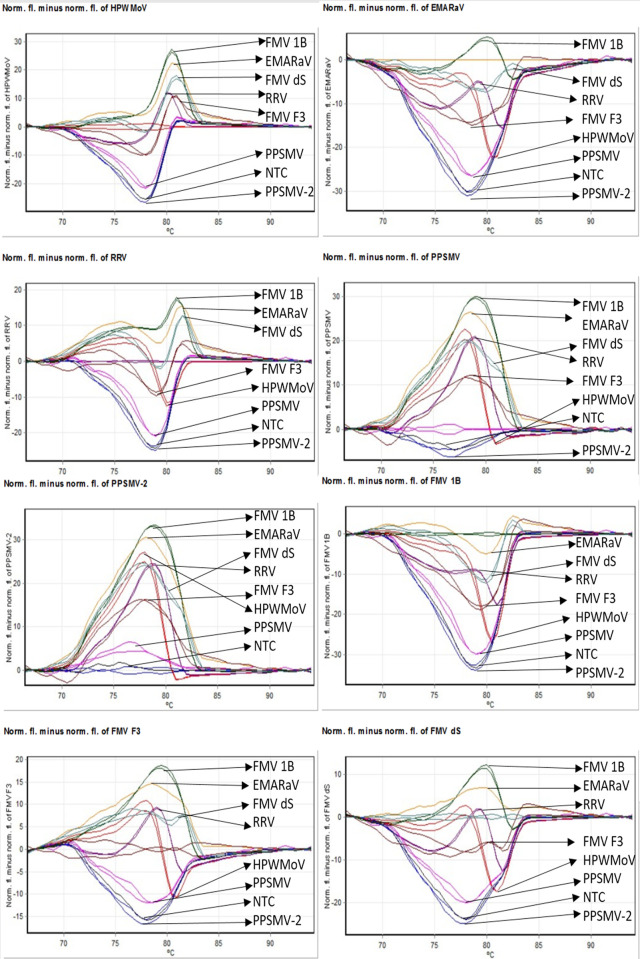
**RT-qPCR-HRM fluorescence difference graphs calculated subtracting the normalized fluorescence graphs of each virus sample tested, starting from top left:** High Plains wheat mosaic virus (HPWMoV), European mountain ash ringspot-associated virus (EMARaV), rose rosette virus (RRV), pigeonpea sterility mosaic virus 1 (PPSMV1), PPSMV2, fig mosaic virus (FMV) isolate 1B, FMV isolate F3, and FMV isolate dS.

The first multiple detection assay (RT-qPCR-HRM, endpoint RT-PCR) was performed in our laboratory at Oklahoma State University, USA. HPWMoV, RRV, EMARaV, FMV dS, FMV F3, and FMV 1B were detected. PPSMV1 and PPSMV2 were not detected (see discussion for details). Unique patterns for each emaravirus and isolates were seen in the normalized melting plot and replicates. Different emaravirus°Tm ranged from 79.51°C to 81.95°C. The primer-dimers detected in NTC were near 100 bp, clearly separated from the 306–309 bp target products, and their°Tm range varied from ~72°C to ~77°C. The primer dimer products amplified in the PPSMV1 and PPSMV2 reactions are shown close to the NTC pattern (Fig 2A). The second multiple detection assay was performed at CIHEAM (Italy) using endpoint RT-PCR only. All assayed emaraviruses tested positive (FMV isolates dS, 1B and F3, PPSMV1, PPSMV2, EMARaV, HPWMoV, RRV, and RYRSaV). Positive controls (plasmids) containing the diagnostic sequence of RRV (positive control 1) and HPWMoV (positive control 2) also tested positive. No amplification of the expected ~306 bp product was observed in the healthy fig tissue used as a negative control (Fig 2B). In a third assay, HPWMoV isolate 07–961 was successfully detected and amplified. HPWMoV 07–961 showed a different melting peak, 80.95°C, different from the HPWMoV reference positive control and the rest of emaraviruses (Fig 2C, left). The HPWMoV°Tm is 79.51°C (Fig 2A). Similarly, RYRSaV was detected plotting its°Tm at 81.89°C (Fig 2C, right). Fluorescence difference curves [55] for each tested emaravirus were generated (Fig 3). These plots allow further discrimination among samples, especially for those having similar melting patterns i.e. HPWMoV and RRV, and the three FMV isolates (Fig 2).

### Limit of detection assays

**Fig 4:**
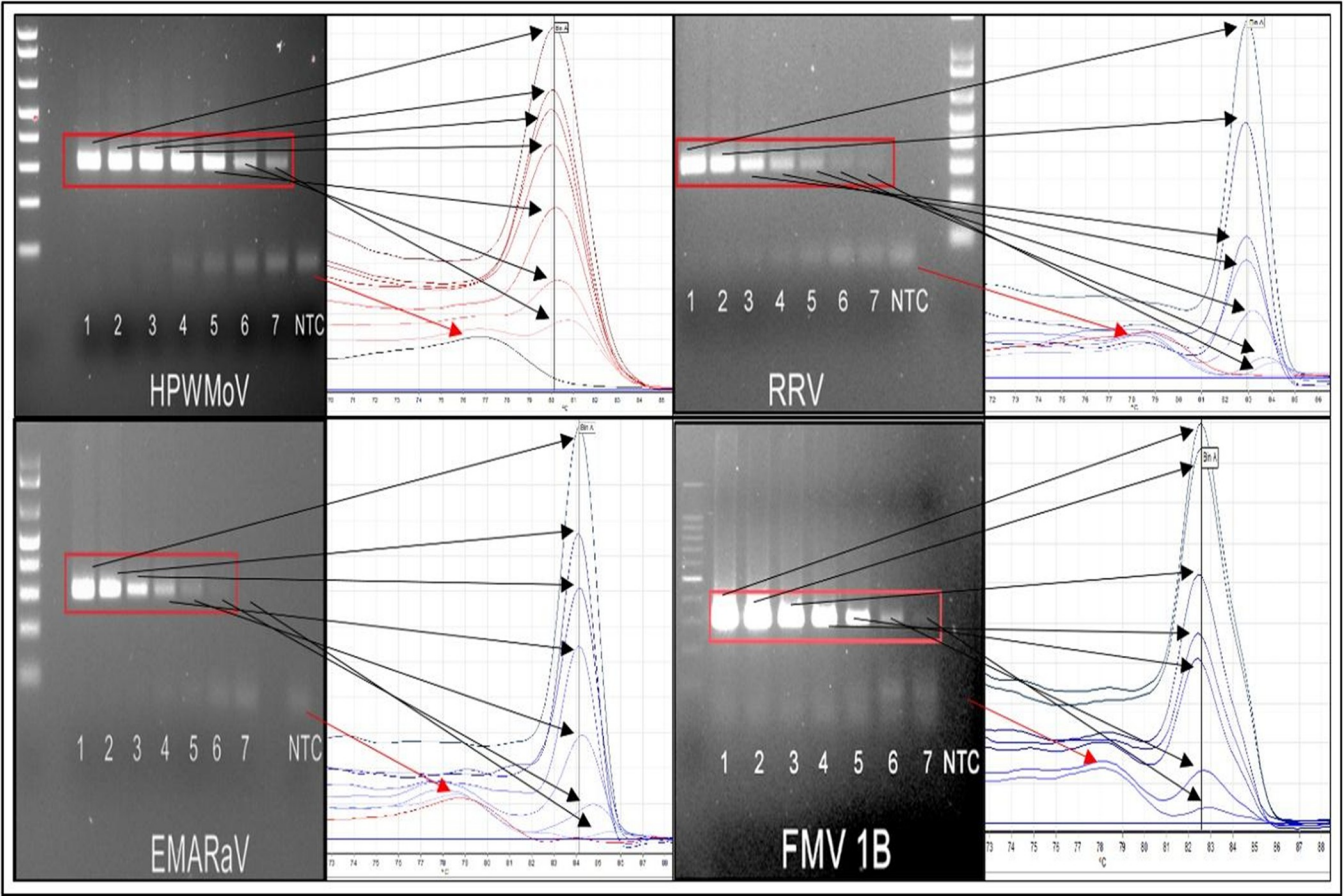
Sensitivity assays of positive controls (plasmids) containing a diagnostic sequence for High Plains wheat mosaic virus (HPWMoV), European mountain ash-ringspot associated virus (EMARaV), rose rosette virus (RRV) and fig mosaic virus (FMV) isolate 1B. The LoD of primers EMARA F7/R8 was 1 fg (endpoint RT-PCR and RT-qPCR-HRM) for all the tested positive controls, except for EMARaV. Sensitivity for EMARaV was 10 fg in RT-PCR, and 1 fg for RT-qPCR-HRM. Plasmid concentrations are 1) 1 ng/μL. 2) 0.1 ng/μL. 3) 10 pg/μL. 4) 1 pg/μL. 5) 0.1 pg/μL. 6) 10 fg/μL. 7) 1 fg/μL. NTC: non-template control (DEPC-treated water).

**Fig 5:**
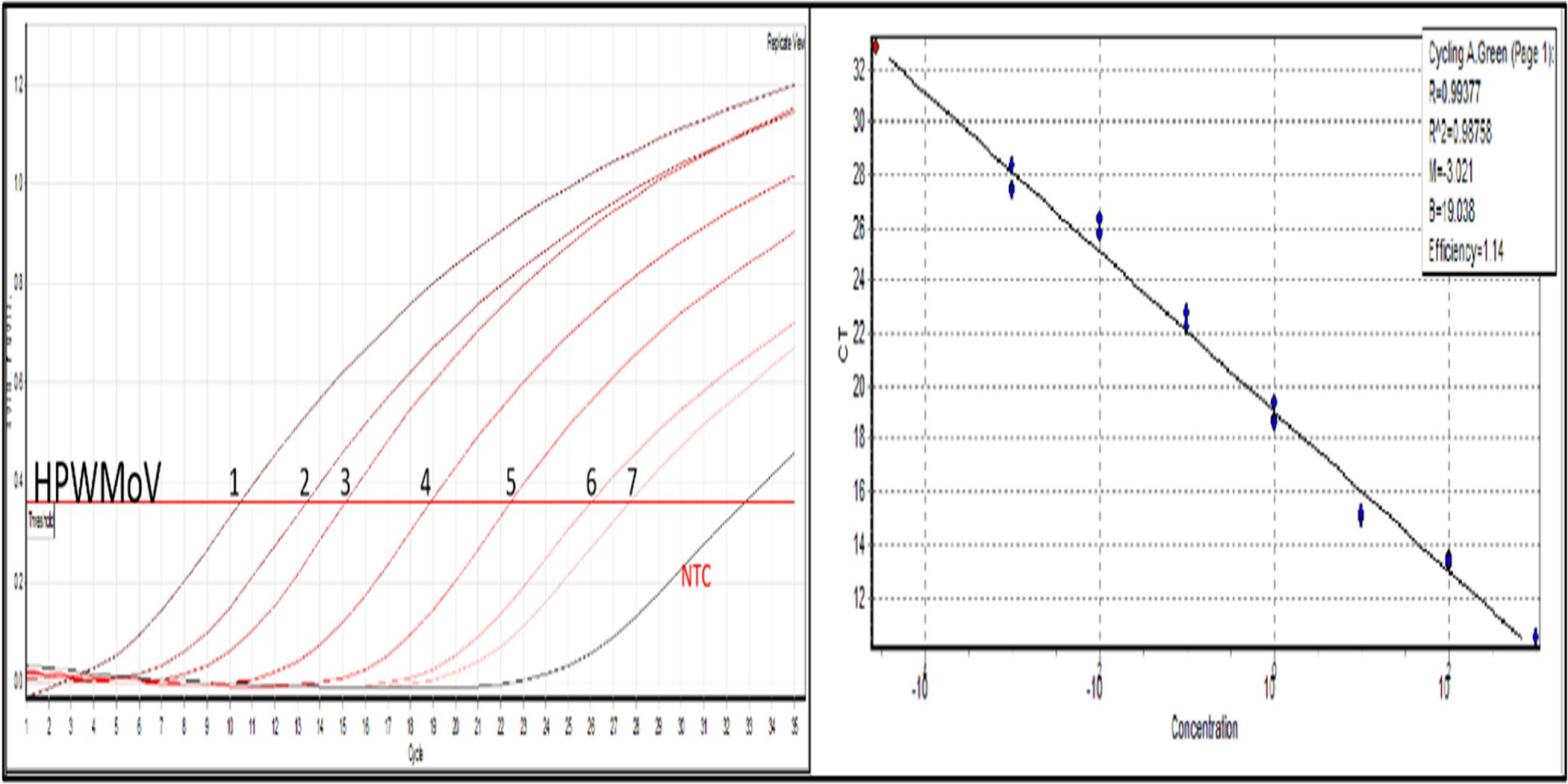
RT-qPCR calibration curve of positive control for High Plains wheat mosaic virus (HPWMoV) using primers EMARA F7/R8. Plasmid concentrations are 1) 1 ng/μL. 2) 0.1 ng/μL 3) 10 pg/μL. 4) 1 pg/μL. 5) 0.1 pg/μL. 6) 10 fg/μL. 7) 1 fg/μL. NTC: non-template control (DEPC-treated water).

The LoD of endpoint RT-PCR and RT-qPCR-HRM with primers EMARA F7/R8 was 1 fg using the positive controls HPWMoV, RRV, and FMV 1B, except for EMARaV (Fig 4). The LoD of RT-PCR for EMARaV was 10 fg. The RT-qPCR-HRM showed sensitivity up to 1 fg for this virus (Fig 4). Primers EMARA F7/R8 perform best at 0.5 mM, and because of their degeneracy primer-dimers formation is visible below the emaravirus amplified products since a high primer concentration is used. However, HRM analysis discriminates primer-dimers from the expected product because of their different°Tm. The concentration of viral cDNA for HPWMoV 07–961 was 5.7 pg determined by the standard curve (Fig 5). The LoD of endpoint RT-PCR and RT-qPCR-HRM assays were 10 fg and 1 fg, respectively using cDNA from HPWMoV 07–961 infected plant tissue (S2 Fig), which was equivalent to the detection limit of the reference positive controls. Interestingly, the HRM analyses showed melting curve patterns shifting towards a larger°Tm at DNA concentrations starting from 100 fg to 1 fg (Fig 4 and S2 Fig).

### Specificity assays

**Fig 6:**
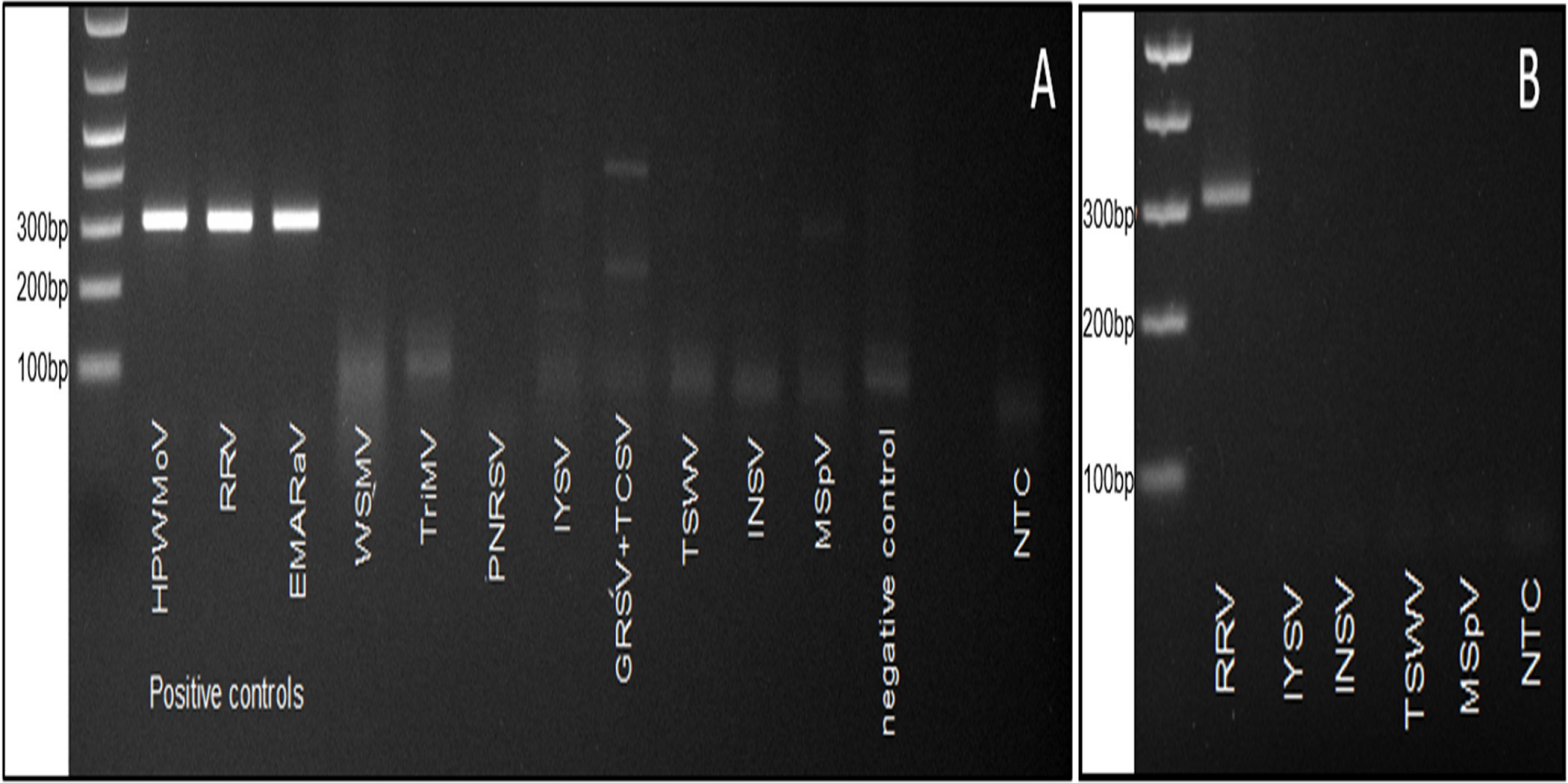
Specificity assay showing inclusivity and exclusivity panels. (A) Specificity assay using GoTAQ polymerase. Inclusivity panel: plasmids (positive controls) for High Plains wheat mosaic virus (HPWMoV), rose rosette virus (RRV) and European mountain ash ringspot-associated virus (EMARaV) were included. Exclusivity panel: cDNA of viruses co-infecting wheat and corn, wheat streak mosaic virus (WSMV) and triticum mosaic virus (TriMV), viruses infecting roses, prunus necrotic ringspot virus (PNRSV), phylogenetically related orthotospoviruses, iris yellow spot virus (IYSV), groundnut ringspot virus (GRSV) mixed with tomato chlorotic spot virus (TCSV), tomato spotted wilt virus (TSWV), impatiens necrotic spot virus (INSV), phylogenetically related tenuivirus, maize stripe virus (MSpV) and cDNA synthesized from healthy wheat-leaves RNA (negative control) were included. (B) Specificity assay using a hot-start polymerase. Inclusivity panel: plasmid for RRV. Exclusivity panel: cDNA for IYSV, INSV, TSWV, MSpV. On both assays, a non-template control (NTC) was included (DEPC-treated water).

Primers EMARA F7/R8 were found specific to the emaraviruses tested. Positive controls HPWMoV, RRV, and RYRSaV were exclusively detected (Fig 6A). Few faint artifacts (non-specific products) were amplified from cDNA of crops commonly infected with emaravirus and phylogenetically related viruses. Still, no amplicons of the expected size were visualized (Fig 6A). The amplification of faint non-specific products in a few of the samples tested may have occurred due to the degeneracy of the primer set. The amplification of these artifacts was avoided using a hot-start polymerase i.e. RRV product in Fig 6B. No bands were amplified from other viruses or the NTC.

### Virus identity and phylogenetic analysis

**Fig 7:**
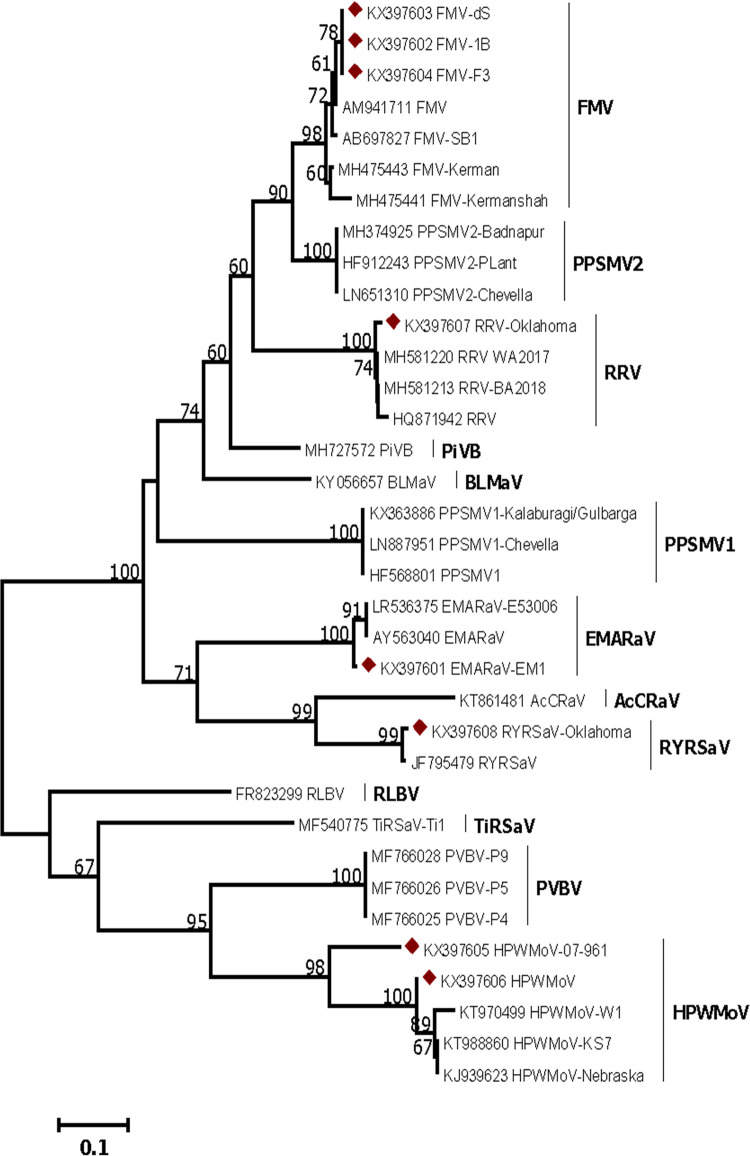
Phylogenetic analysis of protein inferred sequences derived from emaravirus nucleotide sequences amplified with primers EMARA F7/R8 in the first detection assay performed at Oklahoma State University. Red diamond labels are reference emaravirus sequences from this study. The unrooted phylogenetic tree was generated using Maximum Likelihood method with 1000 bootstrap pseudo-replicates as branch support values. Numbers above nodes represent bootstrap values >50. The scale represents the number of substitutions per unit branch length. GenBank accession numbers for each used sequence are provided. Fig mosaic virus (FMV), PPSMV 1 and 2 = pigeonpea sterility mosaic virus 1 and 2, RRV = rose rosette virus, PiVB = Pistacia virus B, BLMaV = blackberry leaf mottle-associated virus, EMARaV = European mountain ash ringspot-associated virus, AcCRaV = Actinidia chlorotic ringspot-associated virus, RYRSaV = redbud yellow ringspot-associated virus, RLBV = raspberry leaf blotch emaravirus, TiRSaV = ti ringspot-associated virus, PVBV = blue palo verde broom virus, HPWMoV = High Plains wheat mosaic virus.

The identity of the emaraviruses tested *in-vitro* was validated by sequencing their RT-PCR products. All the viruses tested had high identity at the nucleotide level (>90%) to homolog accessions deposited in the GenBank, except HPWMoV-07-961, which showed lower identity. EMARaV-EM1 (KX397601) was 96.8% identical to EMARaV (AY563040). The amplified RT-PCR products of FMV isolates 1B, dS, and F3 (Genbank accessions KX397602, KX397603, and KX397604,) were 94.6%, 94.6%, and 94% identical respectively to FMV (AM941711), and showed 98% identity among each other. The RT-PCR product of HPWMoV-07-961 (KX397605) showed 74.4% identity compared to HPWMoV-W1 (KT970499). The HPWMoV reference positive control (KX397606) had 96.4% identity to the other three reference isolates of HPWMoV: GG1 (KT988869), KS7 (KT988860), and Nebraska (KJ939623). The alignment between the two isolates of HPWMoV (07–961 and reference positive control) showed 77.3% nucleotide identity between each other. RRV-Oklahoma 1 (KX397607) had 97.32% identity with two isolates of RRV: WA2017 (MH581220) and BA2018 (MH581213) from California, USA. RYRSaV-Oklahoma (KX397607) had a 93.3% identity to RYRSaV (JF795479). For the construction of the ML phylogenetic tree of the emaravirus RdRp gene, LG+G+I [86], the best model of protein evolution was used (Fig 7). Three major clade groups formed. Clade one: FMV, PPSMV2, RRV, PiVB, BLMaV, and PPSMV1. The second clade is formed by EMARaV, AcCRaV, and RYRSaV, and the third clade is made by RLBV, TiRSaV, PVBV, and HPWMoV. FMV-1B, ds, and F3 clustered together as expected and HPWMoV 07–961 forms a monotypic lineage branching out of the HPWMoV clade. A BLASTX search showed that the 302 bp RdRp sequence (without 5’ non-complementary sequence) of HPWMoV-07-961 had a maximum identity of 78% with a homolog protein belonging to HPWMoV GenBank accession number AML03207.

### Prediction of melting temperature profiles

uMelt^SM^ was used to plot predicted melting curves *in-silico*. Predictions used the expected RT-PCR product sequences, in other words, the RT-PCR diagnostic sequences of the different emaraviruses tested *in-vitro* including the primer sequences (S3 Fig). Table 2 shows a side-by-side comparison among *in-silico* predicted°Tm and the actual°Tm obtained *in-vitro*. Table 2 includes the GC % content of the diagnostic sequences of the emaraviruses tested *in-vitro* as well as the adjusted°Tm predicted *in-silico* using the cooperativity parameter (σ) selected to 0.184407. When the predicted HRM°Tm was calculated without adjusting σ, the *in-silico*°Tm differed 1.86–3.51°C from the actual *in-vitro*°Tm. However, when σ was adjusted, the difference between°Tm *in-silico* and *in-vitro* was substantially reduced. The higher the GC content, the higher the°Tm, although a few°Tm values were larger than expected (Table 2).

**Table 2:** GC content, *in-vitro*, and *in-silico* melting temperatures of different tested emaraviruses. *In-vitro* is the actual melting temperature (°Tm) after HRM. *In-silico* are the predicted°Tm calculated by uMelt^SM^. Number 1 are°Tm with σ as default, number 2 are the predicted°Tm adjusted with σ = 0.184407.

		Melting temperature (°Tm,°C)				
*Emaravirus* member	GC content (%)	*in-vitro*	Prediction (*in-silico*)			
			1	diff	2 (adjusted)	diff
EMARaV	36.9	81.95	79.9	2.05	81.6	0.35
RRV	35.0	80.58	78.7	1.88	80.8	-0.22
FMV 1B	36.9	81.68	78.7	2.98	82.3	-0.62
FMV F3	35.9	81.31	78.8	2.51	81.8	-0.49
FMV ds	36.9	82.31	78.8	3.51	82.4	-0.09
HPWMoV	33.3	79.56	77.7	1.86	79.6	-0.04
07–961	34.6	80.93	79	1.93	80.9	0.03
RYRSaV	35.0	81.89	78.6	3.29	80.8	1.09

diff are°Tm *in-vitro* minus 1 or 2

## Discussion

This study explores and addresses software-assisted prediction of melting curves of RT-qPCR products *in-silico* (predicted) which were compared with *in-vitro* (actual) obtained data using the conserved RdRp partial region of emaraviruses. For this purpose, a primer set compatible with two chemistries, endpoint RT-PCR and RT-qPCR-HRM, was designed and tested. The results of the melting temperatures obtained for emaraviruses are shown in Table 2. The prediction of HRM plots was calculated by uMelt^SM^, which facilitated the selection of the diagnostic region and primers *in-silico*. Testing expected amplicon sequences (including its primer sequences) *in-silico* by uMelt^SM^ facilitated assembling RT-qPCR with HRM, allowed saving time during assay design, and avoided repeated experiments of HRM *in-vitro* searching for the optimal diagnostic region. Previously, [56, 57] reported endpoint RT-PCR methods for the detection of several emaraviruses, this research further contributes knowledge-seeking improvement and contribution on broad detection of virus species within *Emaravirus*.

Primers EMARA F7/R8 uniquely detected emaraviruses and did not amplify false positives after testing six phylogenetically related viruses (orthotospoviruses and tenuiviruses) and three viruses frequently found co-infecting wheat and rose (Fig 6A). Weak non-specific artifacts were initially amplified in endpoint RT-PCR in some samples. These artifacts were avoided using a hot-start polymerase, which allowed sensitivity, reliability, and specificity (Fig 6B). The potential formation of artifacts was expected because the putative degeneration of the two oligo sequences was detected *in-silico*. The amplified RT-qPCR-HRM products were distinguishable from weak non-specific artifacts due to differences in°Tm: 72–77°C for artifacts, in contrast to°Tm over 78°C for emaraviruses (Fig 4).

The multi-emaravirus detection assay was repeated in two locations. Five out of the seven emaraviruses tested positive *in-vitro* in our lab (Oklahoma State University) by RT-qPCR-HRM. PPSMV 1 and 2 tested negative (Fig 2A), which did not allow repeatability for these two viruses, in the inter-laboratory comparison. However, HPWMoV, RRV, RYRSaV, FMV (isolates ds, 1B, and F3), and EMARaV (isolate EM1) detection was repeatable in the two different laboratories. Considering the same cDNA of PPSMV 1 and 2 was used (Fig 2B) in both labs and that PPSMV 1 and 2 were detected at CIHEAM, Italy; the following reasoning may explain this discrepancy. The cDNA of PPSMV 1 and 2 tested at Oklahoma State University may have degraded during intercontinental non-refrigerated handling and shipping. Also, a low titration of the viruses in the original infected tissue [58] may have contributed.

The LoD of the assay varied between 10 fg to 1 fg. EMARaV and HPWMoV 07–961 were detected up to 10 fg by endpoint RT-PCR and up to 1 fg by RT-qPCR-HRM. Whereas the rest of the tested emaraviruses were detected to 1 fg in both endpoint RT-PCR and RT-qPCR-HRM. The oligonucleotide thermodynamic features contributing to good sensitivity are i) the addition of a custom-designed 5`G/C flap to the EMARA R8 primer [47, 52, 59, 60] which balances the GC content and°Tm of the primer set and improves the LoD [47]. ii) A higher primer pair°Tm (~66–68°C) that optimized the reaction with°Ta between 45°C and 53°C. The low°Ta may be due to the insertion of inosines in degenerate positions, and mismatches between the target sequence and the primer, which leads to a decrease in the optimum°Ta of the set [61, 62]. iii) A larger primer length (28 to 35 nucleotides) allowed for increased primer specificity and target sensitivity [63].

Sequencing of amplicons and BLASTn searches confirmed the identity of all the emaraviruses tested in this study. All sequences show a nucleotide identity higher than 90% within species, except for HPWMoV 07–961 which has 74% and 78% identity at nucleotide and protein levels respectively if compared with other HPWMoV isolates. According to [64], HPWMoV isolate 07–961 is another related wheat curl mite-transmitted emaravirus member that belongs to a species different from HPWMoV, which can also cause HPD. An antiserum prepared to HPWMoV 07–961 specifically reacted only to it [64]. The current species demarcation for *Emaravirus* rules a 25% difference in the amino acid sequences of RdRp, GP, or NC [30]. Additional sequencing studies of HPWMoV 07–961 are needed to determine if the virus represents a distinct *Emaravirus* species. HPWMoV 07–961 clustered with HPWMoV isolates, but branched out from the HPWMoV clade and formed a monotypic lineage (Fig 7). The diagnostic data contributed corroborates HPWMoV 07–961 is a distinct emaravirus as reported by [64]. However, additional complete alignment of genomes to include HPWMoV 07–961 and HPWMoV isolates is needed to clarify if the virus represents a distinct *Emaravirus* species.

The obtained diagnostic emaravirus sequences were also used to generate a phylogenetic tree by an ML model. This phylogenetic tree corroborated the emaravirus identity of the sequences, and the tree topology was congruent with the literature [10, 12, 30].

Primers EMARA F7/R8 target conserved regions in the *Emaravirus* RdRp [2, 4, 11] and allowed HRM discrimination, which is possible because of the existing variability among the different emaravirus sequences that are framed between the two primers. This variability is corroborated by the different°Tm among emaraviruses and HRM curve patterns generated in the analysis (Figs 2A, 2C and 3, S2 Fig). Also, previous reports support small amplicons do not only provide successful discrimination among samples in HRM analysis [55]. According to [65], the uniqueness of the melting curve patterns of the targeted amplicons and the melting temperatures are mainly determined by GC content, sequence length, and nucleotide composition. In this study, a relationship between the GC content and°Tm is observed in Table 2. All amplicons had a similar length (306–309 bp) and yet have different°Tm.

The interpretation of HRM results is to be made considering all three HRM results: i) the normalized data derived from plots of raw data as shown in Figs 2 and 3. ii) the Low-Resolution Melt derivative plot (-dF/dT against°Tm in°C) in S3 Fig and Fig 3) the minus difference graphs derived from the normalization data in Fig 3. Fig 2 shows clear differences guided by colored arrows, i.e. EMARaV (yellow) and three isolates of FMV (1B/dark green, F3/brown, and dS/light green), which correspond to°Tm differences of 0.27°C, 0.64°C, and -0.36°C respectively. These differences were confirmed in the minus difference graphs (Fig 3). The minus difference graph shows the normalized and progressive lack of fluorescence plotted against temperature and individually subtracted to each of the virus species being discriminated against. Therefore, once the targeted virus is normalized (subtracted) to itself, it turns zero. EMARaV (Fig 3 top right) is different from the three FMV isolates and the rest of the emaraviruses. HRM was sensitive to discriminate among three isolates of the same virus and within the context of virus species discrimination. Additional observations from HRM plots show nucleotide variability is present in the diagnostic region of FMV.

Interestingly, it is also observed the melting curve patterns of the serially diluted target are slightly shifted rightwards away from the°Tm causing a slight variation (increment) of the°Tm of the less concentrated cDNA (i.e. from 100 fg– 1 fg). These increments in melting temperature were detected from pg toward fg (Fig 4 and S2 Fig). This°Tm variation was explained by [66, 67] upon the basis of the°Tm and DNA concentration are inversely correlated. This artifact in the melting temperature pattern (Fig 4 and S2 Fig) can be reduced by increasing the concentration of MgCl_2_. In our experiments, this inverse correlation was attenuated with 4 mM MgCl_2_ in the reaction (Fig 4) and up to 1 pg.

The cooperativity parameter (σ) was adjusted to better concert *in-silico* and *in-vitro*°Tm values. This adjustment more accurately fits the Blake and Delcourt model [54] and the predicted interaction among the concentrations of MgCl_2_, cDNA, and dye. However, since high mutation rates may be common during replication of some RNA virus species, the assumption of Tm as a single species descriptor during diagnosis is discouraged. Single or dual point mutations occurring in the target may affect the melting curve pattern of the amplicon in the RT-qPCR-HRM assay. Yet, discriminatory tendencies and differences among species will continue to be evident and can be corroborated using biological repeats of the RT-qPCR-HRM assays, a broad number of plant specimens, and viral reference isolates geographically characterized.

In summary, we explored and discussed parameters and criteria to predict and design sensitive, accurate, and reliable diagnostics compatible with two chemistries RT-PCR and RT-qPCR-HRM applied for detection and discrimination of several emaraviruses. The described method allowed the detection of five emaravirus members and discrimination among isolates of the same virus species. Detection of PPSMV 1 and 2 by our proposed emaravirus assays may need further validation. The herein predicted and described RT-qPCR-HRM has the potential to detect candidate emaraviruses including new species or strains that were tested *in-silico*. The use of uMelt^SM^ for the prediction of Tm of diagnostic genomic sequences allowed detection, precise discrimination, and time gaining during assay design since avoids the need for rounds of HRM tests *in-vitro* or streaming routine electrophoresis. However, mutations occurring during virus replication, circumspect the assumption of°Tm as a single species descriptor during diagnosis. Diagnosticians may face difficult diagnoses if dealing with small HRM°Tm differences. Therefore, predicting PCR-product regions with larger Tm differences among amplicons allows to unmistakably discriminate species.

## Supporting information

S1 Fig Multiple nucleotide sequence alignment of two conserved motifs in the emaravirus RdRp gene where primers EMARA F7/R8 were designed using Primaclade. The alignment of EMARA F7 corresponds to the anti-sense strand of the emaraviruses genome, and EMARA R8 corresponds to the sense strand. The underlined sequence in EMARA R8 shows a customized 5’ non-complementary sequence. Stars above the alignment indicate conserved positions. EMARA F7 and EMARA R8 align to the nucleotide regions 4532–4563 and 4830–4803, respectively, in the anti-sense strand genome of the European mountain ash ringspot-associated virus (AY563040). Both primers are in the Bunya_RdRp conserved domain (PFAM: PF04196) in the emaravirus RdRp gene.
(TIF)

S2 Fig Sensitivity assay using cDNA of HPWMoV 07–961 using RT-qPCR-HRM and endpoint RT-PCR formats. The detection limit of both assays was 1 fg using cDNA from infected plant tissue.
(TIF)

S3 Fig Prediction of normalized melting curves for High Plains wheat mosaic virus (HPWMoV), rose rosette virus (RRV), redbud yellow ringspot-associated virus (RYRSaV), HPWMoV 07–961, fig mosaic virus (FMV) isolates F3, EMARaV, FMV isolate 1B and FMV isolate dS. The plot was calculated using uMelt^SM^.
(TIF)

S1 Table Hosts, symptoms, disease, reported genomic RNA segments, and geographic distribution of classified and non-classified emaraviruses [68–86]. (DOCX)

S2 Table Specificity of primers EMARA F7/R8 determined in-silico by Primer-BLAST searches, which does not include the six nt non-complementary sequence (5’flap). The outputs retrieved 13 Emaravirus species. The meaning of the virus acronyms is in the main text.
(DOCX)

S1 Raw images (PDF)
