# Supplementary figures and images for: In-silico prediction of RT-qPCR-high resolution melting for broad detection of emaraviruses
Source: PLoS One. 2023 May 8;18(5):e0272980. doi: 10.1371/journal.pone.0272980 (PMC10166557; doi:10.1371/journal.pone.0272980)

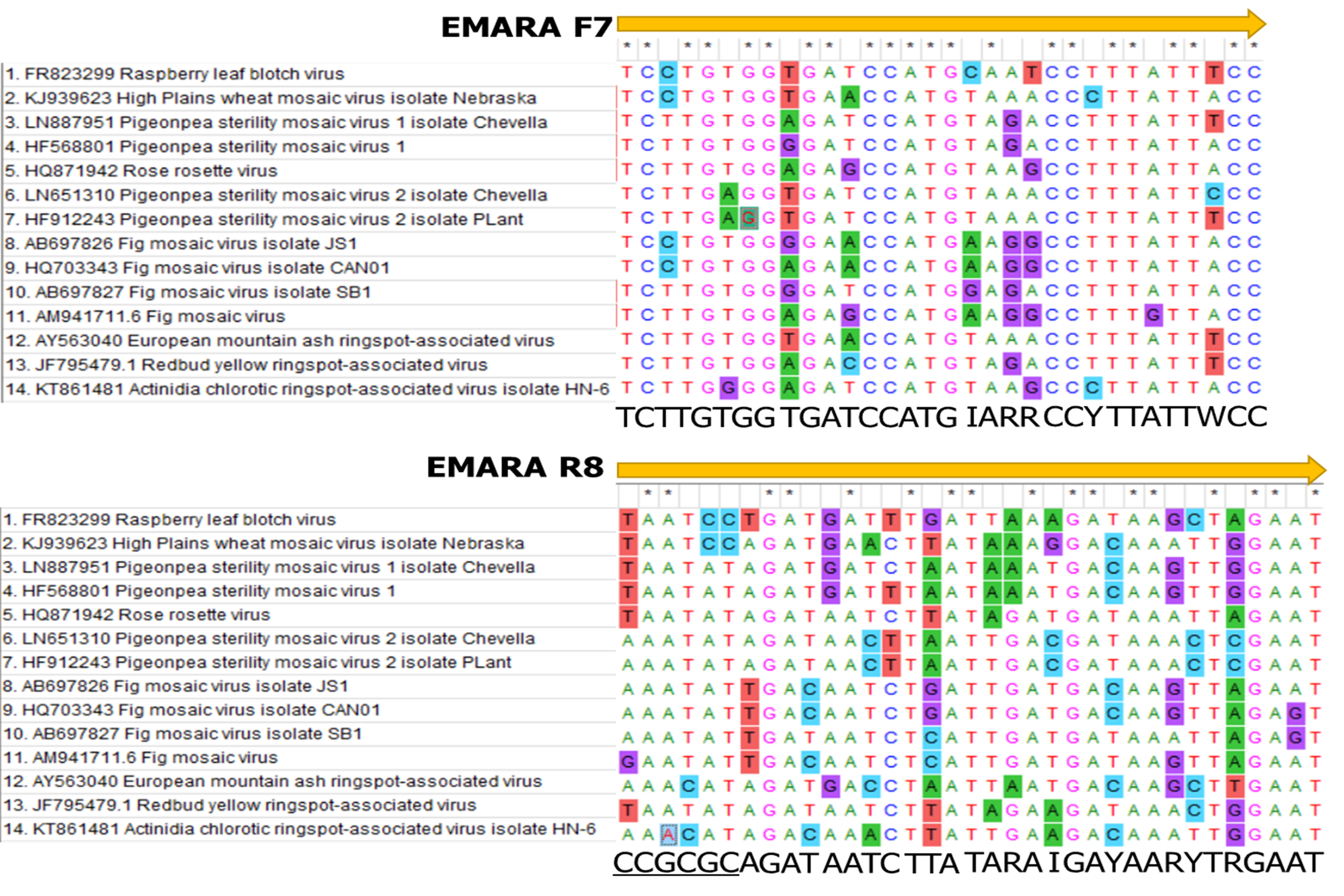

Supplement: S1 Fig — The alignment of EMARA F7 corresponds to the anti-sense strand of the emaraviruses genome, and EMARA R8 corresponds to the sense strand. The underlined sequence in EMARA R8 shows a customized 5’ non-complementary sequence. Stars above the alignment indicate conserved positions. EMARA F7 and EMARA R8 align to the nucleotide regions 4532–4563 and 4830–4803, respectively, in the anti-sense strand genome of the European mountain ash ringspot-associated virus (AY563040). Both primers are in the Bunya_RdRp conserved domain (PFAM: PF04196) in the emaravirus RdRp gene. (TIF) [file pone.0272980.s001.tif]

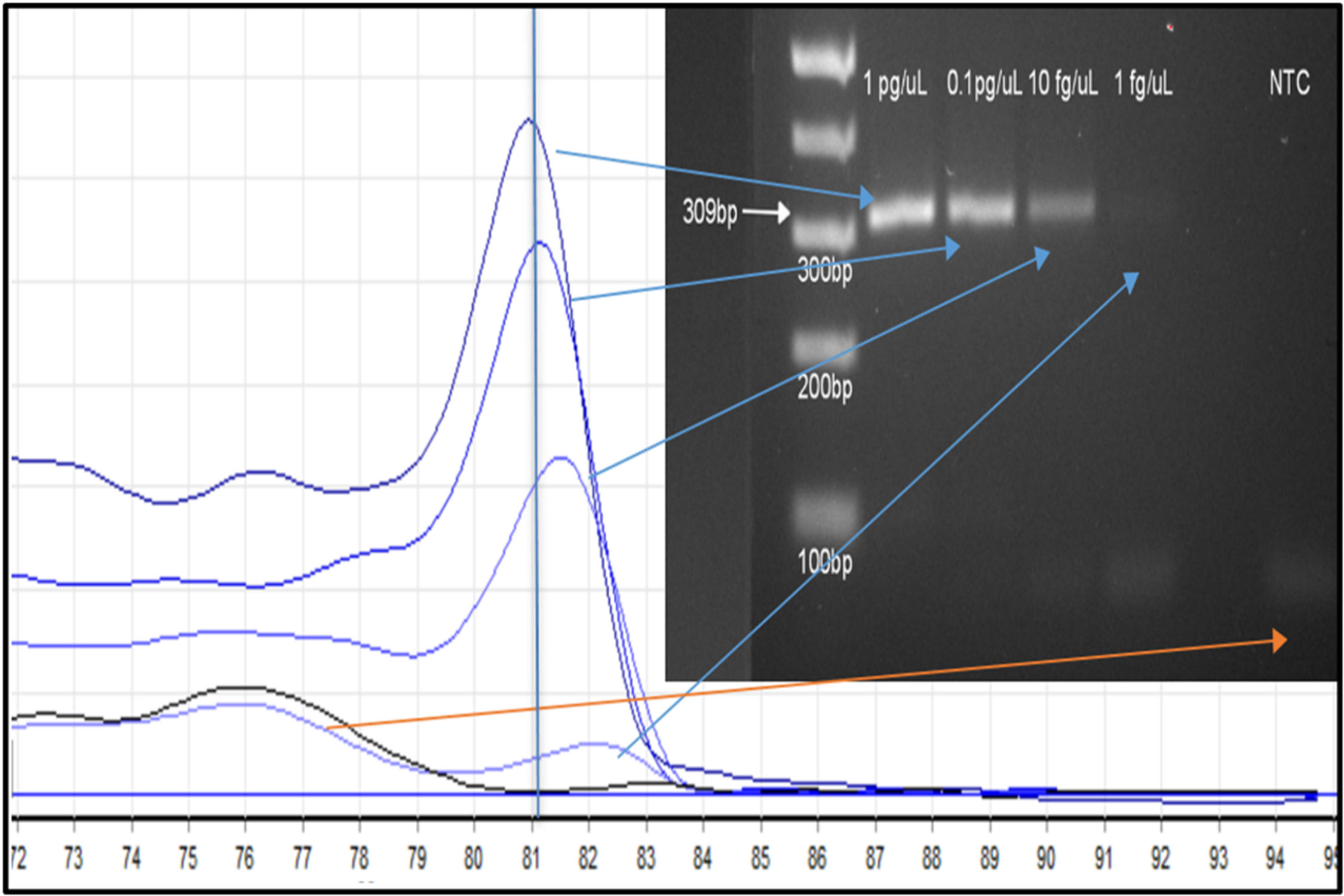

Supplement: S2 Fig — The detection limit of both assays was 1 fg using cDNA from infected plant tissue. (TIF) [file pone.0272980.s002.tif]

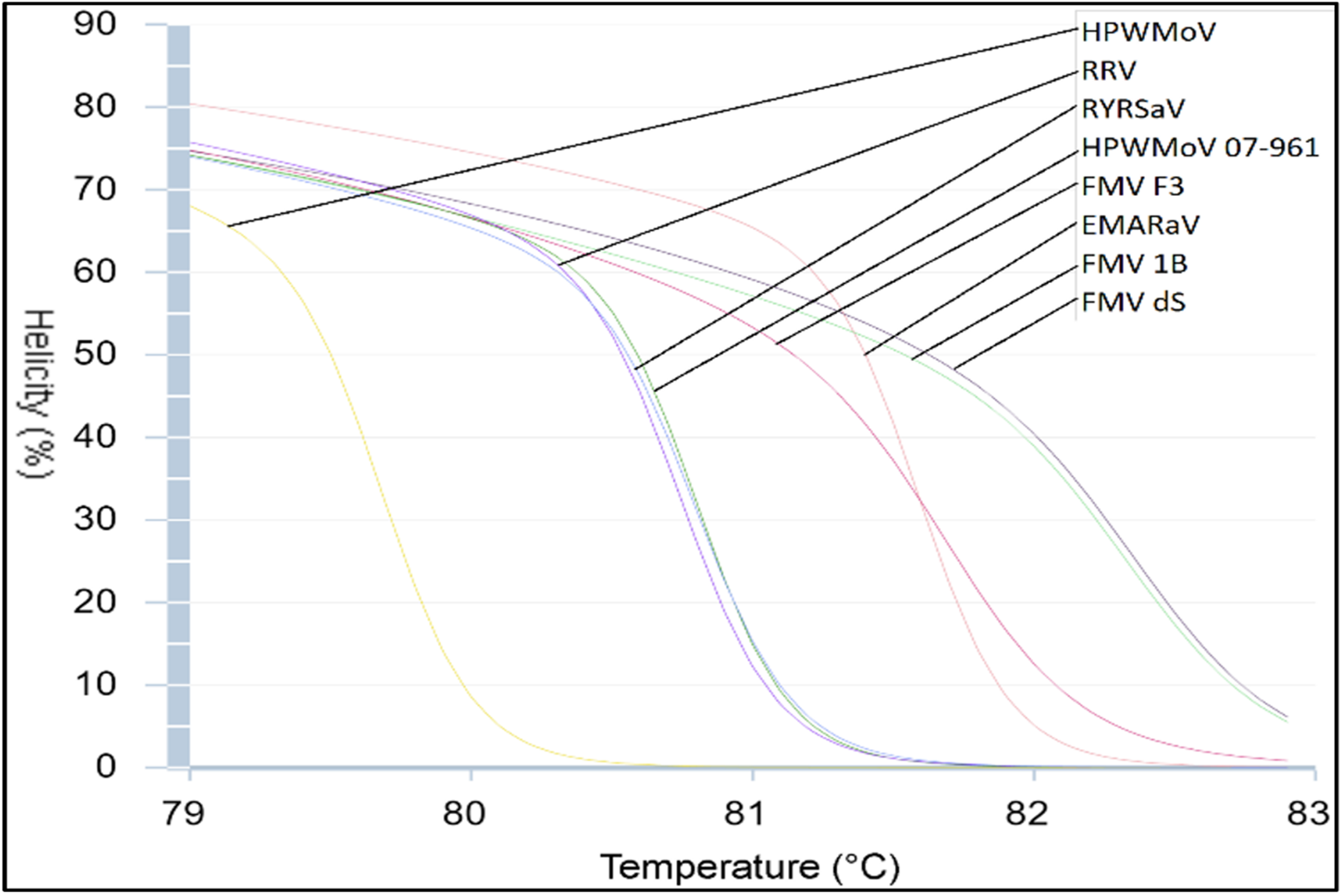

Supplement: S3 Fig — The plot was calculated using uMeltSM. (TIF) [file pone.0272980.s003.tif]
