# Supplementary material for: In-silico prediction of RT-qPCR-high resolution melting for broad detection of emaraviruses
Source: PLoS One. 2023 May 8;18(5):e0272980. doi: 10.1371/journal.pone.0272980 (PMC10166557; doi:10.1371/journal.pone.0272980)
Supplement: S1 Table — (DOCX) [file pone.0272980.s004.docx]

**Table S1.** Hosts, symptoms, disease, reported genomic RNA segments, and geographic distribution of classified and non-classified emaraviruses.

|  |  |  |  |  |  |  |  |  |  |
| --- | --- | --- | --- | --- | --- | --- | --- | --- | --- |
| **No.** | **Emaravirus** | **Disease** | **Symptoms** | **Hosts** | **Presence** | **ICTV Status** | **Segments Reported** | **Notes** | **References** |
| 1 | European mountain ash ringspot-associated virus (EMARaV) | Ringspot disease of European mountain ash trees | Mottling and chlorotic ringspots | *Sorbus aucuparia*, *S. intermedia*, *Aronia melanocarpa* and *Amelanchier* spp. | Europe and Asia | Classified | Six | *S. aucuparia*: Europe-Asia. *S. intermedia*: Sweden.  *A. melanocarpa*: The Czech Republic. *Amelanchier* spp.: Germany. | [1, 68, 69, 70, 71, 72] |
| 2 | Fig mosaic virus (FMV) | Fig mosaic disease (FMD) | Different levels of severity of chlorotic ringspots and mosaics. | Fig and *Cyclamen persicum* Mill | Cosmopolitan | Classified | Six | Other viruses, including closterovirids, have been found in FMD-affected trees and their role in FMD is unclear. | [3, 73, 74, 75] |
| 3 | Rose rosette virus (RRV) | Rose rosette disease (RRD) | Witches broom (rosette type) growth, excessive thorniness, discolored and  distorted growth | Landscape roses | USA, Canada | Classified | Seven | RRV affects aesthetics of roses, degrading their value,  and in severe cases, killing plants in a period of three to five years. RRD threats a $194 million/year industry (USDA, 2010) | [4, 36, 76, 77] |
| 4 | Raspberry leaf blotch virus (RLBV) | Leaf blotch disease in raspberry | Yellow blotching and twisting of leaves, distortion of leaf margins | Raspberry | Europe | Classified | Eight |  | [5, 58, 78, 79] |
| 5 and 6 | Pigeonpea sterility mosaic virus 1 (PPSM1) and PPSMV2 | Sterility mosaic disease (SMD) | Leaf size reduction, severe stunting, severe or mild mosaics and sterility | Pigeonpea | India | Classified | PPSMV1: Five PPSMV2: Six | Single infections of PPSMV and PPSMV2, or a co-infection of both viruses are found. SMD accounts for losses of 300 millions US dollars a year in India | [6, 10, 80, 81] |
| 7 | High Plains wheat  mosaic virus (HPWMoV) | High Plains disease (HPD) | Severe stunting, yellowing and mosaic | Sweet corn, wheat | USA, Australia, Argentina (South America) | Classified | Eight | It can co-infect plants with wheat streak mosaic virus and triticum mosaic virus. The three viruses cause HPD. HPD can cause up to 100% yield loss. HPWMoV is formerly known as High Plains virus or wheat mosaic virus. | [8, 82, 83, 84, 85] |
| 8 | Redbud yellow ringspot-associated virus (RYRSaV) | Redbud yellow ringspot disease | Vein-clearing, yellow ringspots, and oak leaf pattern on leaves. Sometimes tree decline is noticed. | Redbud (*Cercis spp.*) | Arkansas, USA | Classified | Five | Some infected plants are symptomless. | [11] |
| 9 | Blackberry leaf mottle-associated virus (BLMaV) | Blackberry yellow vein disease (BYVD) | Leaf mottling, chlorotic ringspots and curved midrib | Blackberry | USA | Classified | Five | At least ten other viruses are associated to BYVD. The disease affects fruit quality, yield and may cause plant decline. | [13] |
| 10 | Actinidia chlorotic ringspot-associated virus (AcCRaV) | No clearly defined | Ringspots, vein yellowing, and chlorotic spots | Kiwifruit (*Actinidia* spp.) | China | Classified | Five | AcCRaV was found co-infecting kiwifruit trees with Actinidia virus A (AcVA),  AcVB, citrus leaf blotch virus (CLBV) and apple stem grooving virus (ASGV). | [12] |
| 11 | Pistacia virus B (PiVB) | No clearly defined | No clearly defined | Pistachio (*Pistacia*spp.) | Turkey | Classified | Seven | No clear association of symptoms with infection of PiVB, although virus was found in symptomatic tissue. | [14] |
| 12 | Blue palo verde broom virus (PVBV) | Witches’ Broom Disease | Dense clusters of short, flexible, thornless branches with stunted leaves, and branch dieback | Blue palo verde tree (*Parkinsonia florida)* | Arizona, USA | Classified | Four | PVBV genome was partially characterized. | [15] |
| 13 | Ti ringspot-associated virus (TiRSaV) | Ti ringspot disease | Chlorotic ringspots | Ti (*Cordyline aqManse*) | Hawaii, USA | Classified | Five | TiRSaV can be found co-infecting ti plants with velariviruses Cordyline virus 1 (CoV-1), CoV-2, CoV-3 and CoV-4. | [16] |
| 14 | Jujube yellow mottle- associated virus (JYMaV) | Jujube yellow mottle disease | Yellow mottling and fruit malformation | Jujube (*Ziziphus jujuba* Mill.) | China | Classified | Six | Mottling, yellowing and distortion on leaves.  Fruits are malformed, discolored and can present necrotic areas | [17] |
| 15 | Lilac chlorotic ringspot-associated virus (LiCRaV) | No clearly defined | Chlorotic ringspots and  mottling on leaves | Lilac *(Syringa vulgaris L.)* | China | Classified | Five |  | [22] |
| 16 | Aspen mosaic-associated virus (AsMaV) | Mosaic disease | Leaf mottle, yellow blotching and chlorosis along veins | Aspen trees (*Populus tremula*) | Finland, Norway, and Sweden | Classified | Five |  | [18] |
| 17 | Alfalfa ringspot- associated virus (ARaV) | No clearly defined | Small chlorotic ringspots on leaves that often coalesce to form larger lesions. | Alfalfa | Australia | Non Classified | Four | ARaV was partially characterized and possesses at least four genomic segments. | [25] |
| 18 | Pear chlorotic leaf spot-associated virus (PCLSaV) | Pear chlorotic leaf spot disease | Small semi-transparent chlorotic spots on leaves | Sandy pear (*Pyrus pyrifolia*) | China | Classified | Five | PCLSaV was found co-infecting pear trees with ASGV and apple stem pitting virus. | [24] |
| 19 | Pea-associated  emaravirus (PaEV) | No clearly defined | No clearly defined | Pea | Germany | Non Classified | Six | PaEV was partially characterized. This virus was found co-infecting pea with pea enation mosaic virus and pea necrotic yellow dwarf virus | [26] |
| 20 | Actinidia emaravirus 2 (AcEV-2) | No clearly defined | No clearly defined, although virus was found in trees with leaf mottle and  chlorosis | Kiwifruit (*Actinidia* spp.) | China | Classified | Six | AcEV-2 was found in co-infections with AcVA, AcVB, CLBV and Actinidia virus 1 | [23] |
| 21 | Perilla mosaic virus (PerMV) | Perilla mosaic disease | Severe mosaic symptoms on leaves | Shiso (*Perilla frutescens* var. *crispa*) | Japan | Classified | Ten | PerMV is a distinct and highly divergent emaravirus. | [30] |
| 22 and 23 | Camellia japonica- associated emaravirus 1 (CjaEV1) and CjaEV2 | No clearly defined | No clear association, but one research group found these viruses may be associated to chlorotic ringspots | *Camellia japonica* and *C. sinensis* L. | Italy and China | Classified | CjaEV1: Nine CjaEV2: Five | Peracchio et al. (2020) and Zhang et al. (2020) characterized in parallel both viruses. The second research group reported five segments for both viruses, while the first research group reported nine and four for CjaEV1 and CjaEV2, respectively. Wu et al. (2020) found CjEV1 associated to leaf ringspots and flower color breaking in *C. sinensis* L. | [20, 21, 86] |
| 24 | Common oak ringspot- associated virus (CORaV) | Ringspot disease in  common oak | Chlorotic ringspots and spots | Common oak (*Quercus robur* L.) | Germany, Sweden and Norway | Non Classified | Five |  | [27] |
| 25 | Maple mottle-associated virus (MaMaV) | No clearly defined | Mottle and leaf deformation | Sycamore maple (*Acer pseudoplatanus*) | Germany | Non Classified | Six |  | [28] |
| 26 | Chrysantemum mosaic- associated virus (ChMaV) | “Mon-mon” disease | Mosaic or yellowish ringspot symptoms on the leaves | Chrysanthemum (*Chrysanthemum morifolium*) | Japan | Non Classified | Seven |  | [29] |

**References** **Table S1.**

68. von Bargen S, Büttner T, Mühlbach H-P, Robel J, & Büttner C. First Report of European mountain ash ringspot-associated virus in *Sorbus aucuparia* in Norway. Plant Dis. 2014; 98(5), 700. Doi: 10.1094/PDIS-09-13-0955-PDN.

69. von Bargen S, Tischendorf M, & Büttner C. First report of European mountain ash ringspot-associated virus in serviceberry (*Amelanchier* spp.) in Germany. New Disease Reports. 2018; 37, 19. Doi: 10.5197/j.2044-0588.2018.037.019.

70. von Bargen S, Dieckmann HL, Candresse T, Mühlbach HP, Roßbach J, & Büttner C. Determination of the complete genome sequence of European mountain ash ringspot-associated emaravirus from *Sorbus intermedia* reveals two additional segments. Arch Virol 2019; 164(7):1937-1941. <https://doi.org/10.1007/s00705-019-04275-0>.

71. Druciarek T, Lewandowski M. & Tzanetakis IE. First report of European mountain ash ringspot-associated emaravirus in *Sorbus aucuparia* in Poland. Plant Dis. 2019; 103(1): 166. Doi: 10.1094/PDIS-05-18-0720-PDN

72. Grimová L, Marek M, Konrady M, & Ryšánek P. Newly identified host range of European mountain ash ringspot-associated virus (EMARaV) and its distribution in the Czech Republic. Forest Pathol. 2015; 45(3), 177–189. <https://doi.org/10.1111/efp.12151>

73. Elbeaino T, Digiaro M, & Martelli GP. RNA-5 and -6, Two additional negative-sense RNA segments associated with Fig mosaic virus. J Plant Pathol. 2012; 94(2), 421–425.

74. Elbeaino T, Marais A, Faure C, Trioano E, Candresse T. & Parrella, G. High-throughput sequencing reveals *Cyclamen persicum* Mill. As a natural host for Fig mosaic virus. Viruses. 2018b; 10(10), 684. Doi: 10.3390/v10120684.

75. Walia JJ, Willemsen A, Elci E, Caglayan K, Falk BW, & Rubio L. Genetic Variation and Possible Mechanisms Driving the Evolution of Worldwide Fig mosaic virus Isolates. Phytopathol. 2014; 104(1), 108–114. Doi: 10.1094/PHYTO-05-13-0145-R.

76. United States Department of Agriculture. 2007 Census of Agriculture, Washington, DC, USA. 2010; 3, 25.

77. Pemberton HB, Ong K, Windham MT, Olsen J, & Byrne DH. What is rose rosette disease? HortScience. 2018; 53(5), 592-595. <https://doi.org/10.21273/HORTSCI12550-17>.

78. Bi Y, Artola K, Kurokura T, Hytönen T, & Valkonen JPT. First Report of Raspberry leaf blotch virus in Raspberries in Finland. Plant Dis. 2012; 96(8), 1231. Doi: 10.1094/PDIS-04-12-0368-PDN.

79. Jevremović D, Leposavić A, & Paunović SA. Genetic diversity of Raspberry leaf blotch emaravirus in red raspberries from Serbia. Spanish J Agric Res. 2019; 17(1), e1004. Doi: 10.5424/sjar/2019171-13861

80. Patil BL, & Kumar PL. Pigeon pea sterility mosaic virus: a legume-infecting Emaravirus from South Asia. Mol Plant Pathol. 2015; 16(8), 775–786. Doi: 10.1111/mpp.12238.

81. Patil BL, Dangwal M, & Mishra R. Variability of Emaravirus species associated with sterility mosaic disease of pigeon pea in India provides evidence of segment reassortment. Viruses. 2017; 9(7), 183. Doi: 10.3390/v9070183.

82. Burrows M, Franc G, Rush C, Blunt T, Ito D, Kinzer K, et al. Occurrence of viruses in wheat in the Great Plains region, 2008. Plant Health Progress. 2009; 10(1).doi:10.1094/PHP-2009-0706-01-RS.

83. Seifers DL, Martin TJ, Harvey TL, Haber S, Krokhin O, Spicer V, et al. Identiﬁcation of variants of the High Plains virus infecting wheat in Kansas. Plant Dis. 2009; 93:1265–1274. Doi: 10.1094/PDIS-93-12-1265.

84. Coutts BA, Cox BA, Thomas GJ, & Jones RAC. First report of Wheat mosaic virus infecting wheat in Western Australia. Plant Dis. 2014; 98(2), 285. Doi: 10.1094/PDIS-03-13-0288-PDN

85. Alemandri V, Mattio MF, Rodriguez SM & Truol G. Geographical distribution and first molecular detection of an *Emaravirus*, High Plains wheat mosaic virus, in Argentina. Eur J Plant Pathol. 2017; 149(3), 743-750. Doi: 10.1007/s10658-017-1207-8.

86. Wu X, Liu J, & Cheng X. First report of Camellia japonica associated emaravirus 1 associated with camellia leaf ringspot and flower color-breaking disease in China. Plant Dis. 2020; 104(12): 3271. Doi:10.1094/pdis-03-20-0657-pdn
