# Supplementary material for: In-silico prediction of RT-qPCR-high resolution melting for broad detection of emaraviruses
Source: PLoS One. 2023 May 8;18(5):e0272980. doi: 10.1371/journal.pone.0272980 (PMC10166557; doi:10.1371/journal.pone.0272980)
Supplement: S2 Table — The outputs retrieved 13 Emaravirus species. The meaning of the virus acronyms is in the main text. (DOCX) [file pone.0272980.s005.docx]

**Table S2.** Specificity of primers EMARA F7/R8 determined *in-silico* by Primer-BLAST searches, which does not include the six nt non-complementary sequence (5’flap). The outputs retrieved 13 *Emaravirus* species. The meaning of the virus acronyms is in the main text.

|  |  |  |  |  |  |
| --- | --- | --- | --- | --- | --- |
| ***Emaravirus*** | **GenBank** | **Product** | ***Emaravirus*** | **GenBank** | **Product** |
| **hit** | **accessions** | **size (bp)** | **hit** | **accessions** | **size (bp)** |
| AcCRaV | KT861481 | 300 | FMV | KC295745 | 300 |
| BLMaV | KY056657 | 300 |  | KC295741 | 300 |
| EMARaV | AY563040 | 300 |  | KC295759 | 300 |
|  | LR536375 | 300 |  | KC295758 | 300 |
|  | KX397601 | 300 | HPWMoV | KT995099 | 303 |
| FMV | KC295749 | 300 |  | KT970499 | 303 |
|  | MH475441 | 300 |  | KT988869 | 303 |
|  | KC295761 | 300 |  | KT988860 | 303 |
|  | KX397604 | 300 |  | KJ939623 | 303 |
|  | KC295760 | 300 |  | KX397606 | 303 |
|  | KC295757 | 300 |  | KX397605 | 303 |
|  | KC295747 | 300 | PiVB | MH727572 | 300 |
|  | MH475443 | 300 | PPSMV1 | LN887951 | 300 |
|  | KX397602 | 300 |  | KX363886 | 300 |
|  | KC295756 | 300 |  | MH374920 | 300 |
|  | AM941711 | 300 |  | HF568801 | 300 |
|  | KC295754 | 300 |  | KX363896 | 300 |
|  | MH475440 | 300 | PPSMV2 | LN651310 | 300 |
|  | MH475438 | 300 |  | HF912243 | 300 |
|  | MH475437 | 300 |  | MH374925 | 300 |
|  | KC295746 | 300 | PVBV | MF766024 | 300 |
|  | MH475442 | 300 |  | MF766025 | 300 |
|  | LT978305 | 300 |  | MF766026 | 300 |
|  | KX397603 | 300 |  | MF766027 | 300 |
|  | KC295751 | 300 |  | MF766028 | 300 |
|  | KC295750 | 300 | RLBV | FR823300 | 303 |
|  | KC295743 | 300 | RRV | MH581220 | 300 |
|  | MH475439 | 300 |  | MH581213 | 300 |
|  | KC295752 | 300 |  | HQ871942 | 300 |
|  | KC295742 | 300 |  | KX397607 | 300 |
|  | AB697826 | 300 | RYRSaV | JF795479 | 300 |
|  | HQ703343 | 300 |  | KX397608 | 300 |
|  | KC295753 | 300 | TiRSaV | MF540775 | 303 |
