# Supplementary material for: In-silico prediction of RT-qPCR-high resolution melting for broad detection of emaraviruses
Source: PLoS One. 2023 May 8;18(5):e0272980. doi: 10.1371/journal.pone.0272980 (PMC10166557; doi:10.1371/journal.pone.0272980)
Supplement: S1 Raw images — (PDF) [file pone.0272980.s006.pdf]

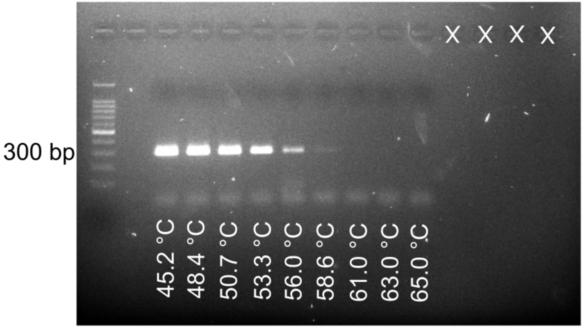

Fig1A. Gradient PCR to optimize annealing temperature for EMARA F7/R8

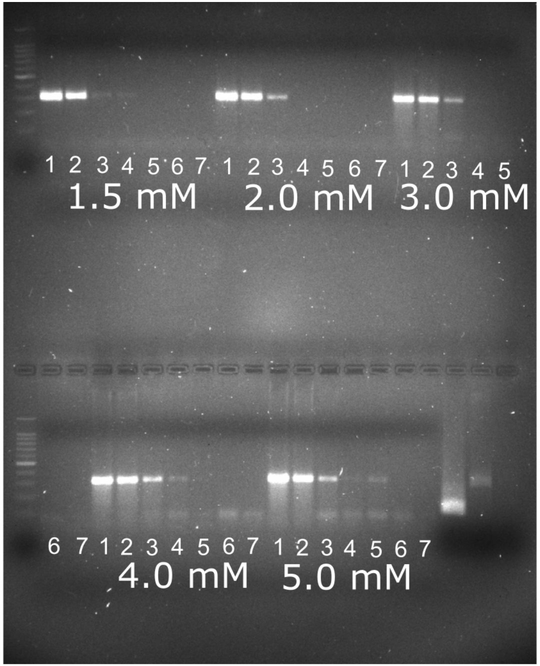

Fig1B. Optimization of magnesium chloride using serially diluted cDNA of HPWMoV

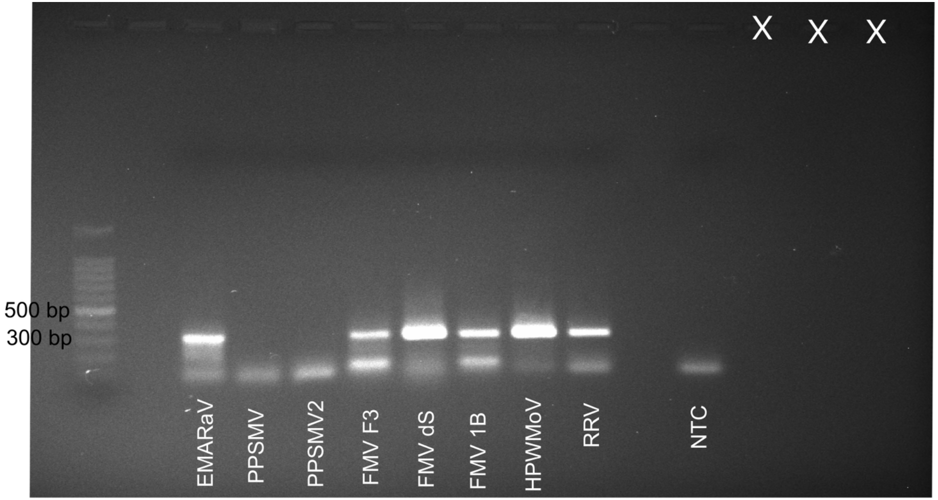

Fig2A. Multiple emaravirus detection of several members that belong to seven Emaravirus species.

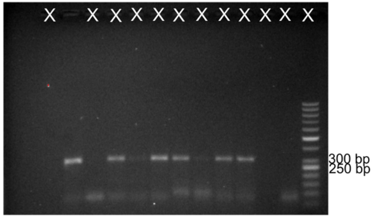

Fig2C-left. Detection of HPWMoV using EMARA F7/R8

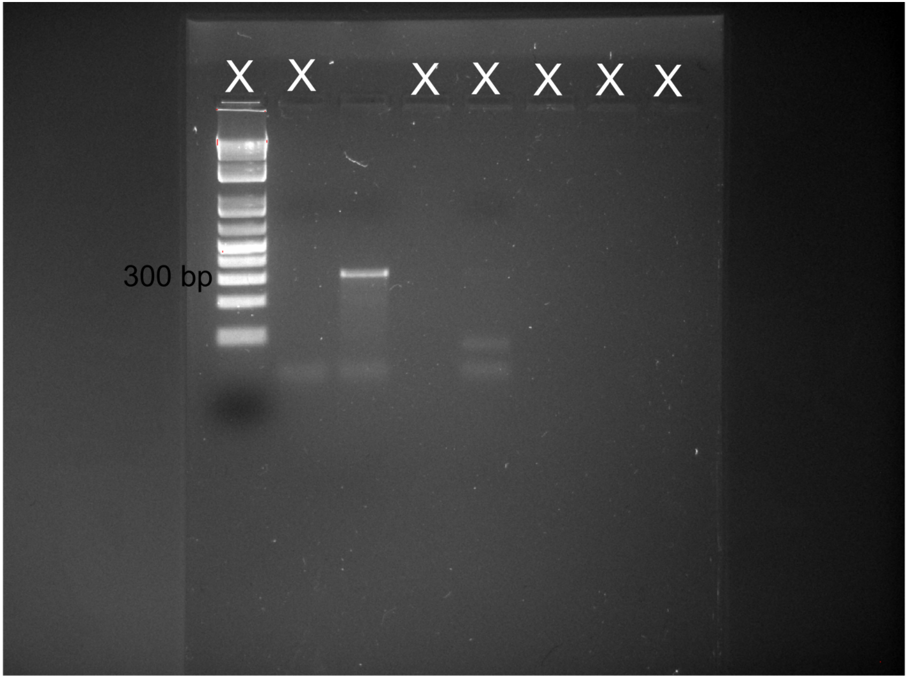

Fig2C-right. Detection of RYRSaV using EMARA F7/R8

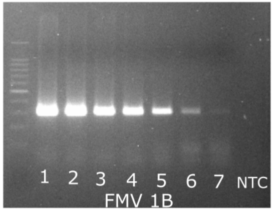

Fig4-bottom-right. Sensitivity of EMARA F7/R8 using RT-PCR assays and serially diluted cDNA of FMV

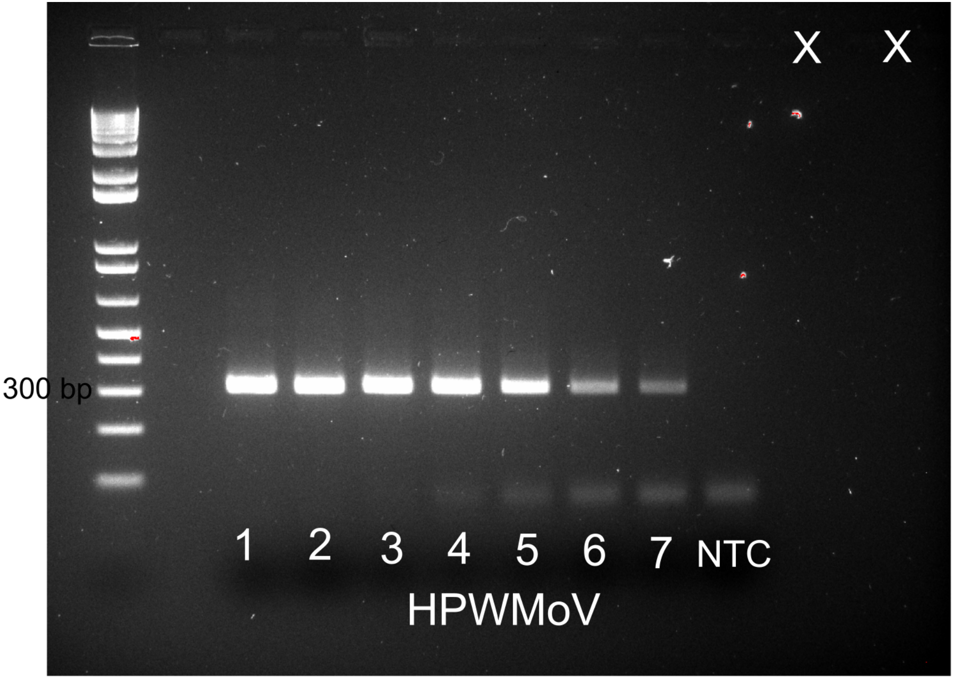

Fig4-top-left. Sensitivity of EMARA F7/R8 using RT-PCR assays and serially diluted cDNA of HPWMoV

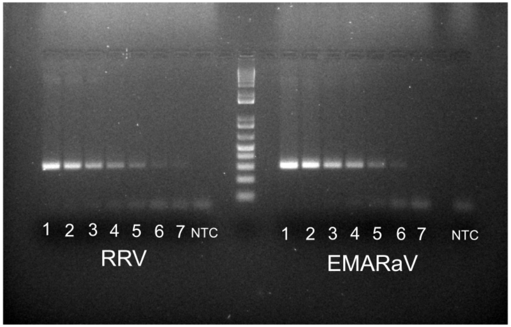

Fig4 (top-right and bottom-left).
Sensitivity of EMARA F7/R8 using RT-PCR asays
and serially cDNA of RRV and EMARaV

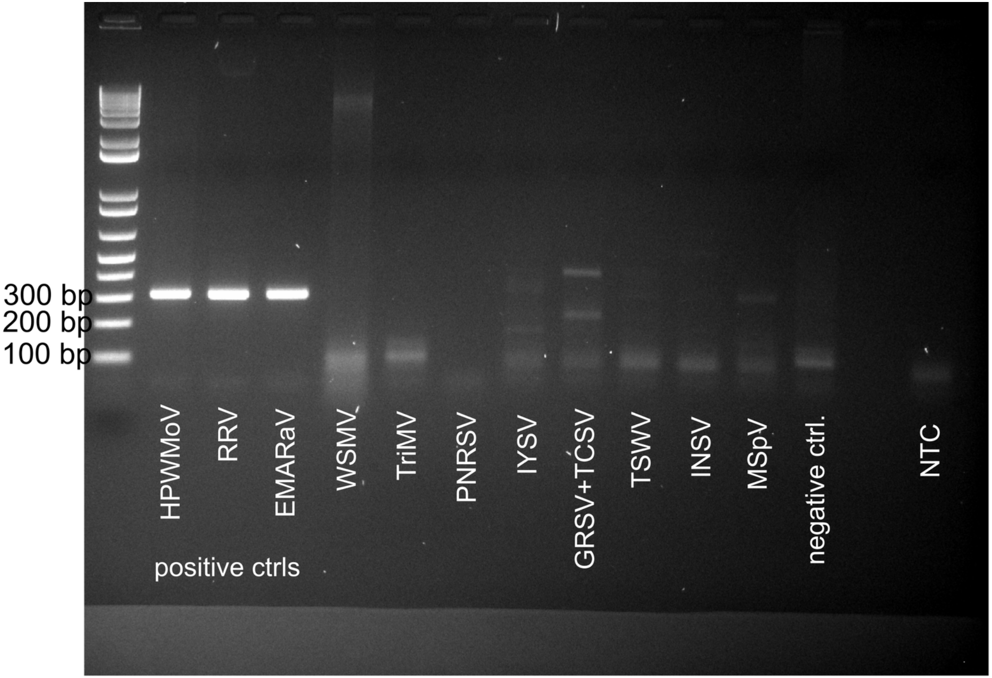

Fig6A. Specificity assay of EMARA F7/R8 using RT-PCR assays

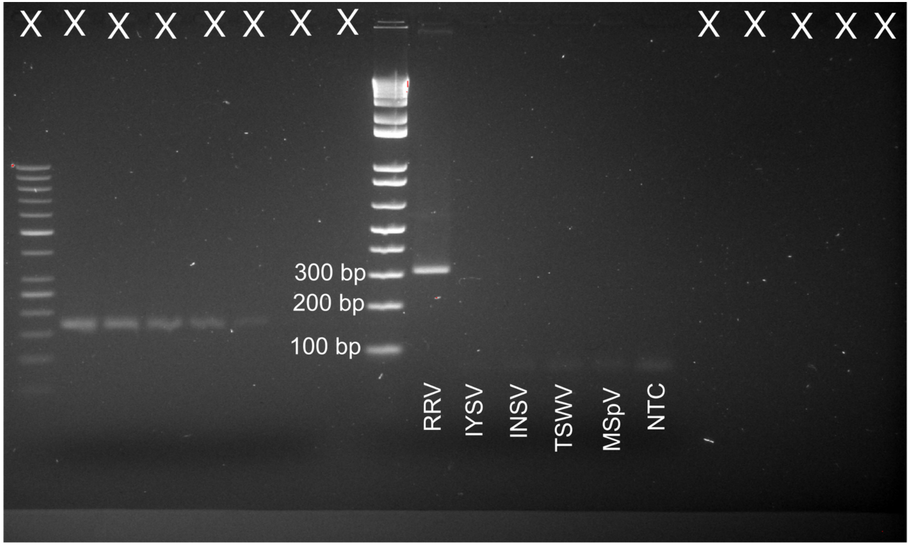

Fig6B. Specificity assays of EMARA F7/R8 using RT-PCR assays with a hot-start polymerase

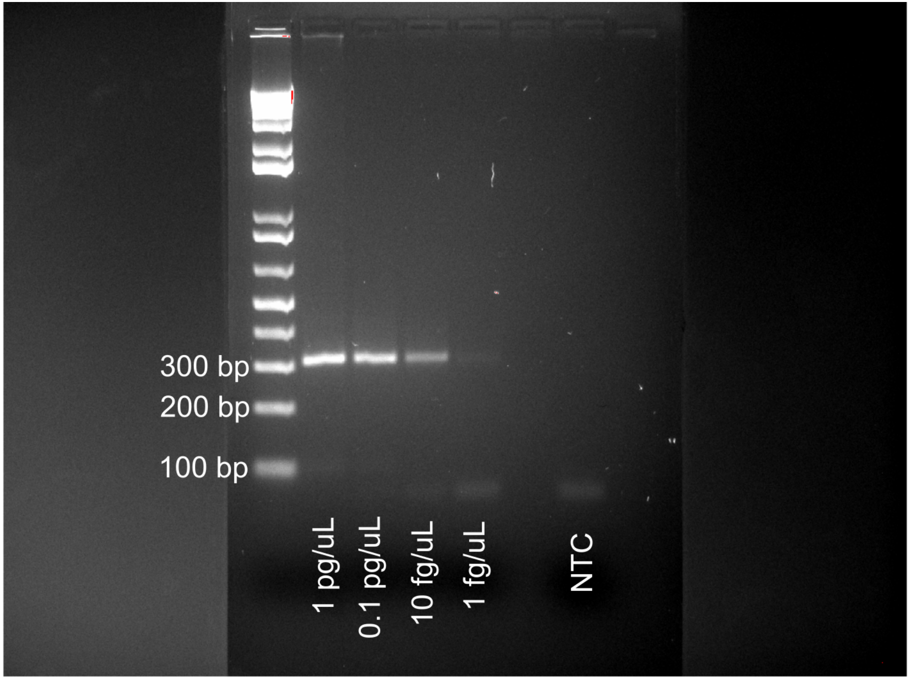

S2-Fig. Sensitivity of EMARA F7/R8 using serially diluted cDNA of HPWMoV 07-961
